# Chemical Variation of Chenpi (Citrus Peels) and Corresponding Correlated Bioactive Compounds by LC-MS Metabolomics and Multibioassay Analysis

**DOI:** 10.3389/fnut.2022.825381

**Published:** 2022-02-23

**Authors:** Mei Yang, Zongde Jiang, Mingchun Wen, Zhenfeng Wu, Minyu Zha, Wen Xu, Liang Zhang

**Affiliations:** ^1^Key Laboratory of Modern Preparation of TCM, Ministry of Education, Jiangxi University of Chinese Medicine, Nanchang, China; ^2^State Key Laboratory of Tea Plant Biology and Utilization, International Joint Laboratory on Tea Chemistry and Health Effects of Ministry of Education, Anhui Agricultural University, Hefei, China; ^3^The Second Clinical College, Guangzhou University of Chinese Medicine, Guangzhou, China

**Keywords:** citrus peel, metabolomics, polymethoxyflavones, biological activity, quantitative

## Abstract

The peel of *Citrus reticulata* “Chachi” (CP) possesses various health-promoting benefits and is not only one of the most famous Chinese herbal medicine, but also an ingredient in fermented foods. In the present study, the effects of storage years (1-, 3-, 4-, 5-, 6-, and 11-years) on the chemical profiling and potential bioactive compounds of CP were compared by metabolomics and *in vitro* bioactivity analysis. With the increase of storage time, the content of hesperidin significantly decreased, but nobiletin, 3,5,6,7,8,3′,4′-heptamethoxyflavone, and tangeretin were increased. Meanwhile, the antioxidant activity of CP was enhanced. Phenolic acids, flavonol glycosides, fatty acids, and alkyl glycosides were marker compounds that were responsible for distinguishing the storage time of CP. Correlation analysis suggested that some polyphenols including quercetin-glucoside, quinic acid, trihydroxydimethoxyflavone, and rutin were potential antioxidant compounds in CP. The dichloromethane and *n*-butanol fractions showed the better antioxidant capacity and inhibitory effects on glucose-hydrolysis enzymes. They mainly contained ferulic acid, nobiletin, 3,5,6,7,8,3′,4′-heptamethoxyflavone, kaempferol, and hesperidin.

## Introduction

Citrus peel (CP) is the dried and mature peel of *Citrus reticulata* Blanco and its cultivars. It is not only a common fruit, but also possesses various health benefits. It has been widely desired by many consumers due to its unique aroma (1). The main production areas of CP are Guangdong, Sichuan, Zhejiang, and Jiangxi Province of China. CP can be divided into common CP and Canton CP according to its plantation origins, where Canton CP is mainly produced in Xinhui City, Guangdong Province. Most studies have shown that CP is a beneficial food with a wide range of health benefits, such as antiinflammation, antiasthmatic, antithrombosis, anticarcinogenesis, and antioxidation activities (2–4). More than hundreds of compounds have been purified and identified in CP, mainly including polymethoxyflavones (PMFs), limonin, and alkaloids (5–7).

Polymethoxyflavones of CP usually exist as the forms of glycosides, and their structures are very similar. These PMFs mainly include sinensetin, 3,5,6,7,8,3',4'-heptamethoxyflavone, 5,7,8,3',4'-pentamethoxyflavone, hesperidin, and nobiletin. Numerous studies have shown that PMFs possess antiinflammatory and antitumor activities, and have been recognized as typical bioactive substances of CP (8, 9). The quantitative analysis of these components have been employed by thin-layer chromatography (TLC), gas chromatography-mass spectrometry (GC-MS), liquid chromatography-diode array detection (LC-DAD), and liquid chromatography-mass spectrometry (LC-MS) (10, 11). Li et al. used high-performance liquid chromatography (HPLC) to quantitatively analyze 6 kinds of PMFs in different commercial CP extracts (12). Fu et al. established a simple and accurate method by using HPLC with dual-wavelength detection to determine the contents of a flavanone glycoside (hesperidin) and 5 kinds of PMFs in CP (13). Camarda et al. quantified flavonoids in citrus herbs by thin layer chromatography (TLC) (14).

It is believed that the quality of CP is improved with storage years (15). Some studies have found that the contents of flavonoids, such as hesperidin, nobiletin, and tangeretin in CP, increased with storage years (16). On the contrary, some studies showed that with the increase of storage years, the content of PMFs decreased (17). However, the comprehensive analysis on the chemical changes of various main and trace metabolites of CP during storing was still unclear so far. Therefore, it is worth deeply investigating the relationship between the chemical profiling changes of CP and its biological activities.

Metabolomics analysis is a potent tool in identifying chemical constituents of foods and plants. Untargeted metabolomics analysis provides dynamic changes in metabolites and could be used to find the difference between the samples, which is limited by the resolution of the analytical instrument. Although, targeted metabolomics is usually used to quantify the differential compounds by optimizing the pretreatment method and the chromatographic and mass spectrometry separation conditions, which can provide a result with good repeatability and sensitivity. However, it is limitedly used to analyze a large number of marker compounds due to the time-consuming methodology (18–21).

In the present study, the effects of storage years (1-, 3-, 4-, 5-, 6-, and 11-years) on the chemical profiling and potential bioactive compounds of CP were compared by metabolomics and *in vitro* bioactivity analysis. The aim of this study was to identify the marker compounds of CP during storage and find the relationship between these compounds and the biological activities of CP, furthermore providing a new strategy of thought of comprehensive utilization of CP.

## Materials and Methods

### Chemicals and Materials

The raw materials of the collected CP samples were fruits of *Citrus reticulata* “Chachi.” All fresh CPs of *Citrus reticulata* “Chachi” were picked from December every year at the same fruit garden of Xinhui District, Guangdong Province of China (latitude, 22.30382; longitude, 113.0634). The processing methods of all CP samples were the same, mainly including cleaning and sun-drying. Six samples have been stored for 1-, 3-, 4-, 5-, 6-, and 11-years (2020–2010, and were named as 1Y, 3Y, 4Y, 5Y, 6Y, and 11Y, respectively). Before analysis, all the samples were stored at room temperature (25°C).

Hesperidin, nobiletin, 3,5,6,7,8,3',4'-heptamethoxyflavone, tangeretin, α-glucosidase, α-amylase (from porcine pancreas), *p*-nitrophenyl-α-d-glucopyranoside (PNPG, biotechnology level, ≥99%), 1,1-diphenyl-2-picrylhydrazyl (DPPH), DNS reagent (mainly including 3,5-dinitrosalicylic acid, NY/T method), phosphate buffer saline (PBS, powder), and total antioxidant capacity (T-AOC) test kits [including 2,2'-Azino-bis(3-ethylbenzothiazoline-6-sulfonic acid) (ABTS) and ferric reducing antioxidant power (FRAP) assays] were purchased from Shanghai Yuanye Biotechnology Company (Shanghai, China). The purities of all chemical standards were ≥98%. Formic acid (MS-grade) and acetonitrile (MS-grade) were purchased from TEDIA Company Inc. (Fairfield, OH, USA). Analytical grade petroleum ether, dichloromethane, ethyl acetate, and *n*-butanol were purchased from Sinopharm chemical reagent Co., Ltd. (Shanghai, China).

### Sample Preparation

To determine the contents of four PMFs in CP with different storage years, six CP samples were crushed into 200 mesh size. In a 50-mL centrifuge tube, 1.00 g aliquot of each CP powder was weighted and 5 ml of methanol solution was added. Then the mixture was extracted for 10 min at room temperature (25°C) with ultrasonic, and sequentially centrifuged for 10 min at 11,963 × g at 25°C. The extraction procedure was repeated once. The supernatants were combined and diluted to 10 ml with methanol. The final extraction solution was filtered through a 0.22-μm microfiltration membrane prior to LC-MS analysis; meanwhile the extracts of CP were mixed equally as quality control (QC). For *in vitro* assays of antioxidant and antihyperglycemic activity, each extract was concentrated using a rotary vacuum evaporator (EYELA N-1100, Tokyo, Japan) at 40°C to remove the methanol and then dissolved in 10 mL distilled water.

To explore the active components in CP, the extract of CP was separated using several organic solvents according to our previous study (22). In brief, 10.00 g CP powder (3-years) was extracted for 30 min at 80°C using 200 mL 75% ethanol (*v*/*v*) by reflux extraction method, then the extract was concentrated by a rotary vacuum evaporator mentioned above, and finally dissolved in 50 mL distilled water. Thereafter, the sample solution was successively extracted three times with an equivalent volume of petroleum ether, dichloromethane, ethyl acetate, and *n*-butanol. Then, the organic solvents of these extracts were removed before redissolving them to 50 mL with distilled water, labeling them as A (petroleum ether extract), B (dichloromethane extract), C (ethyl acetate extract), D (*n*-butanol extract), and E (the remaining aqueous fraction). The solutions (A–E) were filtered *via* a 0.22 μm polyether sulfone membrane prior to LC-MS analysis and *in vitro* biological activities assays.

### Quantitative Analysis of PMFs in CP Samples

To establish the calibration curves of PMFs and determine their contents in CP, an Agilent 1260 HPLC system (Agilent Technologies, Palo Alto, CA, USA) consisting of infinity binary pump, integrated vacuum degasser, autosampler, thermostated column compartment, and diode array detector (DAD) was used. For the separation of the PMFs, an Agilent C_18_ column (250 × 4.6 mm, 5 μm) was used. The HPLC settings were as follows: injection volume, 5 μL; column oven, 30°C; flow rate, 1.0 mL/min; detection wavelength, 330 nm. The mobile phase consisted of distilled water with 0.1% formic acid (A) and acetonitrile (B). The gradient elution was: 0–10 min, 20–20% B; 10–15 min, 20–45% B; 15–17 min, 45–50% B; 17–27 min, 50–50% B; 27–32 min, 16–16% B; 27–30 min, 50–58% B; 30–35 min, 58–65% B; 35–40 min, 65–20% B; 40–45 min, 20% B.

Four kinds of PMFs (hesperidin, nobiletin, 3,5,6,7,8,3',4'-heptamethoxyflavone, and tangeretin) were precisely weighed and dissolved in methanol. Then, the mixed standard solution was diluted to different concentrations (500, 200, 100, 50, 10, and 5 μg/mL) before HPLC analysis.

### Untargeted Metabolomics Analysis

#### LC-MS Analysis

The CP samples were analyzed using an Agilent 1290 LC system (Agilent Technologies, Palo Alto, CA, USA) coupled to a time-of-flight mass spectrometer (Agilent Technologies, Palo Alto, CA, USA). The separation of the chemical compounds was conducted using Waters ACQUITY UPLC HSS T_3_ (2.1 × 100 mm, 1.8 μm). The LC-MS settings were as follows: injection volume, 3 μL; flow rate, 0.08 mL/min; column temperature, 25°C; mobile phase consisted of 0.1% formic acid in distilled water (*v*/*v*, A) and acetonitrile (B), and the gradient elution: 0–2 min, 5–10% B; 2–4 min, 10–15% B; 4–7 min, 15–20% B; 7–14 min, 20–25% B; 14–17 min, 25–50% B; 17–20 min, 50–95% B; 20–25 min, 95–5% B; 25–30 min, 5% B. The mass spectrometer was operated in the negative ionization mode at a resolving power of 20,000 over a full scan range of *m*/*z* 100–1,500 with the following settings: sheath gas temperature, 350°C; nebulizer, 35 psi; gas flow, 8 L/min; gas temperature, 320°C; sheath gas flow, 11 L/min; sheath gas, auxiliary gas, and collision gas are all high purity nitrogen.

#### Data Analysis

Mass spectrometric data were qualitatively analyzed by MassHunter Qualitative Analysis (version B.07.00) software. The MS raw data were further uploaded into MS-DIAL (version 2.90) software for processing, and then the multivariate analysis was conducted by the SMICA-P (14.1, Umetrics, Umeå, Sweden) software. Principal component analysis (PCA) and hierarchical cluster analysis (HCA) were used as unsupervised multivariate analyses to initially classify the data to obtain a general overview of the metabolites. In addition, extra data (storage time) was designated as a Y variable and analyzed statistically by partial least squares (PLS), partial least squares discriminant analysis (PLS-DA), and orthogonally corrected partial least squares discriminant analysis (OPLS-DA). In the OPLS-DA analysis results, the integration of VIP values and *S*-plot results were used to find marker compounds in CP during storage (23).

### Targeted Metabolomics Analysis

To identify the marker compounds between these CP samples and the major compounds in several extraction fractions, QC samples, and extraction fractions were analyzed by LC-Q-TOF-MS. All related parameters were the same as mentioned above. LC-Q-TOF-MS/MS mode was employed to detect the targeted compounds. The collision energies (CE) were set up for the parent ion at *m*/*z* 114.0186 (1.31 min, CE 10 mV), 131.0453 (1.27 min, CE 15 mV), 132.0289 (1.36 min, CE 15 mV), 152.9945 (23.66 min, CE 15 mV), 163.1116 (22.56 min, CE 18 mV), 165.0387 (1.34 min, CE 15 mV), 179.0546 (1.35 min, CE 15 mV), 191.0552 (1.35 min, CE 25 mV), 203.0652 (1.35 min, CE 10 mV), 205.0338 (1.40 min, CE 15 mV), 255.2306 (21.95 min, CE 27 mV), 256.0229 (20.13 min, CE 10 mV), 265.1459 (22.32 min, CE 35 mV), 269.0438 (13.97 min, CE 20 mV), 271.0596 (13.66 min, CE 15 mV), 277.2148 (20.19 min, CE 20 mV), 279.2316 (23.15 min, CE 25 mV), 284.0305 (19.75 min, CE 15 mV), 293.0963 (1.34 min, CE 10 mV), 299.0540 (14.33 min, CE 20 mV), 308.0541 (21.33 min, CE 20 mV), 322.1061 (21.92 min, CE 30 mV), 323.0782 (21.32 min, CE 15 mV), 327.0482 (10.68 min, CE 10 mV), 329.0278 (19.65 min, CE 30 mV), 338.1029 (21.34 min, CE 25 mV), 341.1067 (1.31 min, CE 15 mV), 341.0648 (12.85 min, CE 15 mV), 353.0636 (9.05 min, CE 20 mV), 433.1105 (9.41 min, CE 20 mV), 463.1214 (10.72 min, CE 20 mV), 469.1316 (18.67 min, CE 25 mV), 473.1052 (9.05 min, CE 15 mV), 487.1951 (20.10 min, CE 20 mV), 489.2109 (19.93 min, CE 30 mV), 555.2806 (23.81 min, CE 40 mV), 593.1477 (9.05 min, CE 35 mV), 593.1473 (11.92 min, CE 35 mV), 607.1625 (14.93 min, CE 20 mV), 609.1410 (8.27 min, CE 35 mV), 651.2611 (14.40 min, CE 45 mV).

### α-Glucosidase and α-Amylase Inhibition Assays

The determination of α-amylase and α-glucosidase enzyme inhibition assay followed a previously described method with some modifications (24, 25). Briefly, for the α-glucosidase assay, in a 96-well plate, 50 μL of CP solution with different concentrations, or blank (distilled water) was added, then mixed with 100 μL of 1 U/mL α-glucosidase (dissolved in 0.1 M PBS, pH 6.8), whereafter the mixed solution was incubated for 10 min at 37°C. Finally, 50 μL of 5 mM PNPG (dissolved in 0.1 M PBS, pH 6.8) was added to each well and incubated for 5 min at 37°C before the absorbance was read at 405 nm using a SpectraMax M2 multimode microplate reader. Results were presented as percent inhibition according to the formula below.


$$\begin{array}{l} \text{Glucosidase inhibition rate }=\;\frac{{\text{A}}_{\text{control}}\;-({\text{A}}_{\text{test}}-\;{\text{A}}_{\text{blank}})}{{\text{A}}_{\text{control}}}\\ \;\times 100, \end{array}$$

where *A*_control_ is the absorbance of sample without CP extract, *A*_test_ is the absorbance of sample containing CP extract, and *A*_blank_ is the absorbance of sample containing CP extract, but without enzyme solution.

For the α-amylase assay, in a centrifuge tube, 500 μL of the CP extract with different concentrations, or blank (distilled water) was mixed with 300 μL of 13 U/mL α-amylase solution (dissolved in 0.02 M PBS, pH 6.8), and then the mixture was incubated for 5 min at 37°C. Then, 300 μL of 1% soluble starch (previously dissolved in water and boiled for 10 min) was added to each tube and incubated for 25 min at 37°C. Finally, 500 μL of DNS reagent was added and the mixed solution was heated in water bath for 5 min at 100°C. Thereafter, the heated mixture was cooled to room temperature (25°C) and diluted to 5 ml with distilled water, and then the absorbance was read at 520 nm using a SpectraMax M2 Multimode microplate reader. Results were presented as percent inhibition according to the formula below.


$$\begin{array}{l} \text{Amylase inhibition rate }=\;\frac{{\text{A}}_{\text{control}}\;-({\text{A}}_{\text{test}}-{\text{A}}_{\text{blank}})}{{\text{A}}_{\text{control}}}\\ \;\times \;100,\text{where} \end{array}$$

*A*_control_ is the absorbance of sample without CP extract, *A*_test_ is the absorbance of sample containing CP extract, and *A*_blank_ is the absorbance of the sample containing CP extract, but without enzyme solution.

### Determination of Antioxidant Activities

The T-AOC assay of CP samples was analyzed using the DPPH, FRAP, and ABTS methods (13, 26–28). For the DPPH assay, in a centrifuge tube, 400 μL of the CP extract with different concentrations, or blank (distilled water) was mixed with 400 μL 56 μg/mL DPPH solution, and then the mixture was incubated in the dark for 30 min at room temperature (25°C). Finally, 200 μL of the mixture was taken out and its absorbance was recorded at 517 nm using a SpectraMax M2 Multimode microplate reader. Results were presented as percent free radical scavenging rate according to the formula below,


$$\begin{array}{l} \text{DPPH scavenging rate }=\;\frac{{\text{A}}_{\text{blank}}-{\text{A}}_{\text{test}}}{{\text{A}}_{\text{blank}}}\;\times \;100 \end{array}$$

ABTS^+^ assay of CP was determined using the test kit. Briefly, in a 96-well plate, 7 μL of CP extract with different concentrations, or blank (distilled water) was mixed with 280 μL ABTS^+^ solution, and then the mixture was incubated at room temperature for 6 min. Thereafter the absorbance of the mixture was recorded at 734 nm using a SpectraMax M2 Multimode microplate reader. The ABTS^+^ inhibition rate was calculated using the equation as below.


$$\begin{array}{l} \text{ABT}{\text{S}}^{+}\text{ inhibition rate }=\;\frac{{\text{A}}_{\text{blank}}-{\text{A}}_{\text{test}}}{{\text{A}}_{\text{blank}}}\times 100 \end{array}$$

FRAP assay of CP was also determined using the test kit. Briefly, in a 96-well plate, 10 μL CP extract with different concentrations, or control (distilled water) was mixed with 88 μL FRAP solution, and then incubated for 30 min at 37°C. The absorbance of the mixture was recorded at 593 nm using a SpectraMax M2 multimode microplate reader. For calculating the antioxidant activity of samples, the Fe^2+^ standard solution was diluted to several concentrations and then treated as the same procedure mentioned above to establish the calibration curve (*Y* = 0.5567^*^X + 0.0792, *R*^2^ = 0.9994).

### Data Processing and Statistical Analysis

All samples were analyzed in triplicates and the results are expressed as mean value plus or minus standard deviation (*n* = 3). The linear correlation among antioxidants, antihyperglycemic activity, and the chemical composition of the samples were analyzed by SIMCA-P 14.1 software. *P* < 0.05 was considered significant by one-way ANOVA analysis.

## Results and Discussion

### Contents of PMFs in CP With Different Storage Years

**Figure 1:**
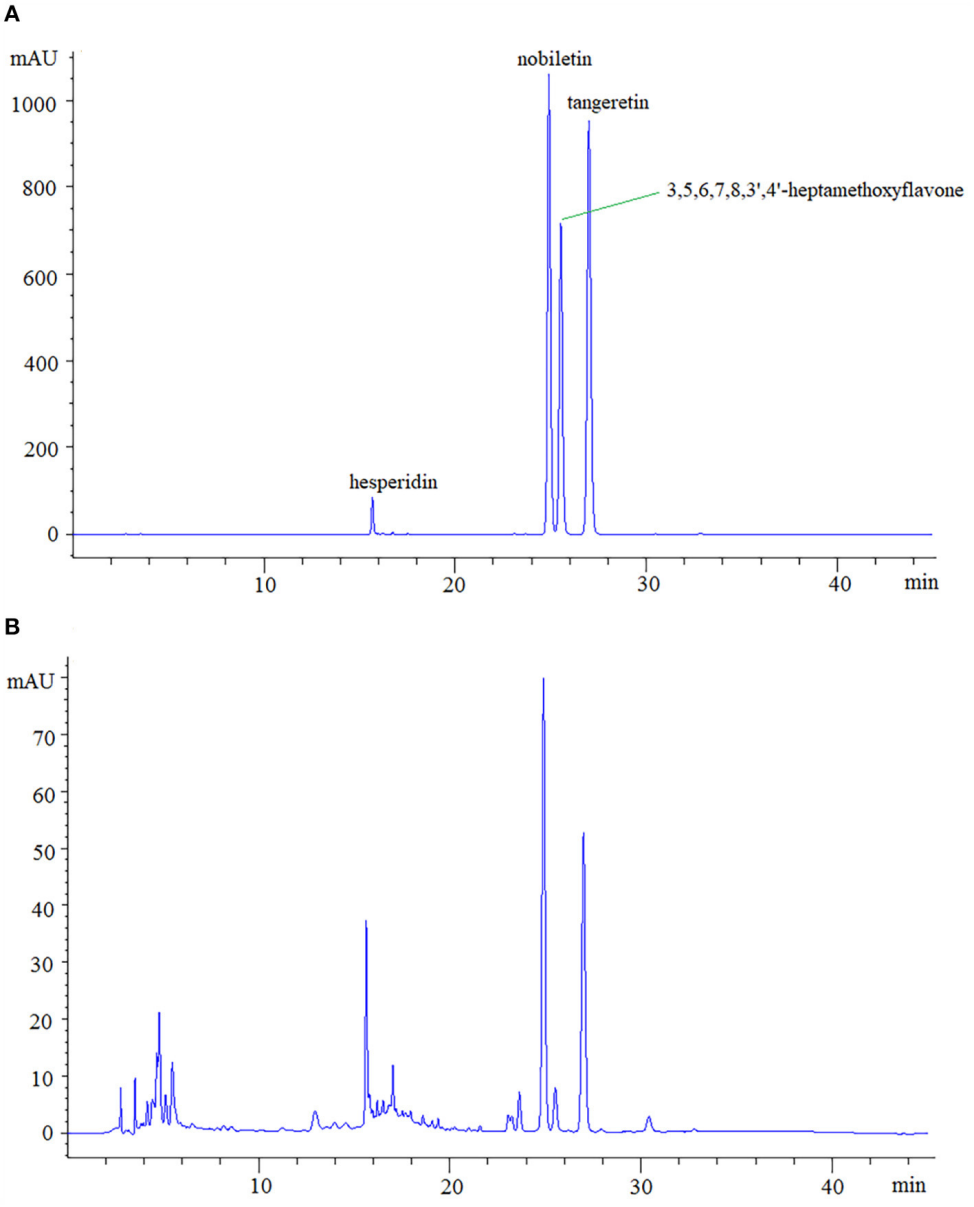
The HPLC spectrum of four analytes **(A)** and CP samples **(B)**.

According to a previous study, PMFs were the main components in CP (29). Thus, in the present study, we determined the contents of 4 kinds of PMFs (including hesperidin, nobiletin, 3,5,6,7,8,3',4'-heptamethoxyflavone, and tangeretin) in CP by HPLC during storage. As shown in Figure 1A, 4 kinds of PMFs had good selectivity inreverse phase elution system under optimized chromatographic conditions, thereinto, hesperidin had the shortest retention time compared to other standards due to its glycoside structure, which was consistent with the results of previous studies (30, 31). In addition, these four PMFs also had good selectivity as shown in Figure 1B, and most of the other unknown peaks were qualitatively analyzed by LC-MS.

Calibration curves of these PMFs were established with six levels of concentration and covered ranges at 5–200 μg/mL. The linearity and correlation coefficient (r) of the calibration curves are presented in Table 1. The correlation coefficient of these calibration curves was ≥0.9999, which indicated that these calibration curves of PMFs had a good linearity.

**Table 1:** The calibration curves of four flavonoids by HPLC.

**Compounds**	***t_***R***_* (min)**	**Calibration curves**	**r**	**Linear range (μg/mL)**
Hesperidin	15.57	Y = 1.39*X + 0.13	0.9999	5–200
Nobiletin	24.81	Y = 19.12*X – 38.27	0.9999	5–200
3,5,6,7,8,3',4'-Heptamethoxyflavone	25.41	Y = 13.59*X – 27.48	0.9999	5–200
Tangeretin	26.88	Y = 20.68*X – 40.10	0.9999	5–200

After a period of storage, CP possesses a unique flavor and some health benefits (32). Meanwhile, there were some changes of chemical constituents in CP during storage, which may affect some biological activities. As shown in Table 2, using the calibration curves of the main compounds, the changes of four flavonoids in CP with different storage years were determined. The contents of hesperidin, nobiletin, 3,5,6,7,8,3',4'-heptamethoxyflavone, and tangeretin were higher in CP after 4-years of storage than other years of storage. However, as the storage time increases, the contents of these four PMFs marginally decreased. It indicated that CP needed at least 4-years of storage and it could be stored for a long time (31). Thereinto, hesperidin markedly changed during storage, for instance, its content mildly increased after a short time of storage ( ≤ 4-years of storage) while it significantly decreased after a long time of storage. After 11-years of storage, the content of hesperidin was 8.98 ± 1.80 mg/g, even lower than that (13.05 ± 0.71) after 1-year of storage. However, further studies are needed to identify the transformation mechanism of these PMFs in CP during storarge and the effects of these changes on biological activities (33).

**Table 2:** The contents of four flavonoids in CP with different storage years (1-, 3-, 4-, 5-, 6-, and 11-year) (mg/g).

**Compounds**	**1-year**	**3-year**	**4-year**	**5-year**	**6-year**	**11-year**
Hesperidin	13.05 ± 0.71^ab^	15.44 ± 1.02^a^	16.07 ± 0.50^a^	10.26 ±1.77^b^	6.32 ± 2.43^b^	8.98 ± 1.80^b^
Nobiletin	3.56 ± 0.04^cd^	4.03 ± 0.09^c^	5.50 ± 0.11^a^	3.32 ± 0.03^d^	4.02 ± 0.39^c^	4.68 ± 0.38^b^
3,5,6,7,8,3',4'-Heptamethoxyflavone	0.62 ± 0.01^c^	0.63 ± 0.01^c^	0.98 ± 0.01^a^	0.64 ± 0.02^c^	0.75 ± 0.05^b^	0.81 ± 0.04^b^
Tangeretin	2.64 ± 0.02^b^	3.14 ± 0.07^a^	3.34 ± 0.06^a^	2.47 ± 0.03^b^	2.71 ± 0.20^b^	3.08 ± 0.16^a^

*Different letters in superscript in a same row indicate level of chemical compound with statistically significant difference (p < 0.05), by one-way ANOVA (Tukey' multiply comparisons test)*.

### LC-MS Based Untargeted Metabolomics Analysis Results

**Figure 2:**
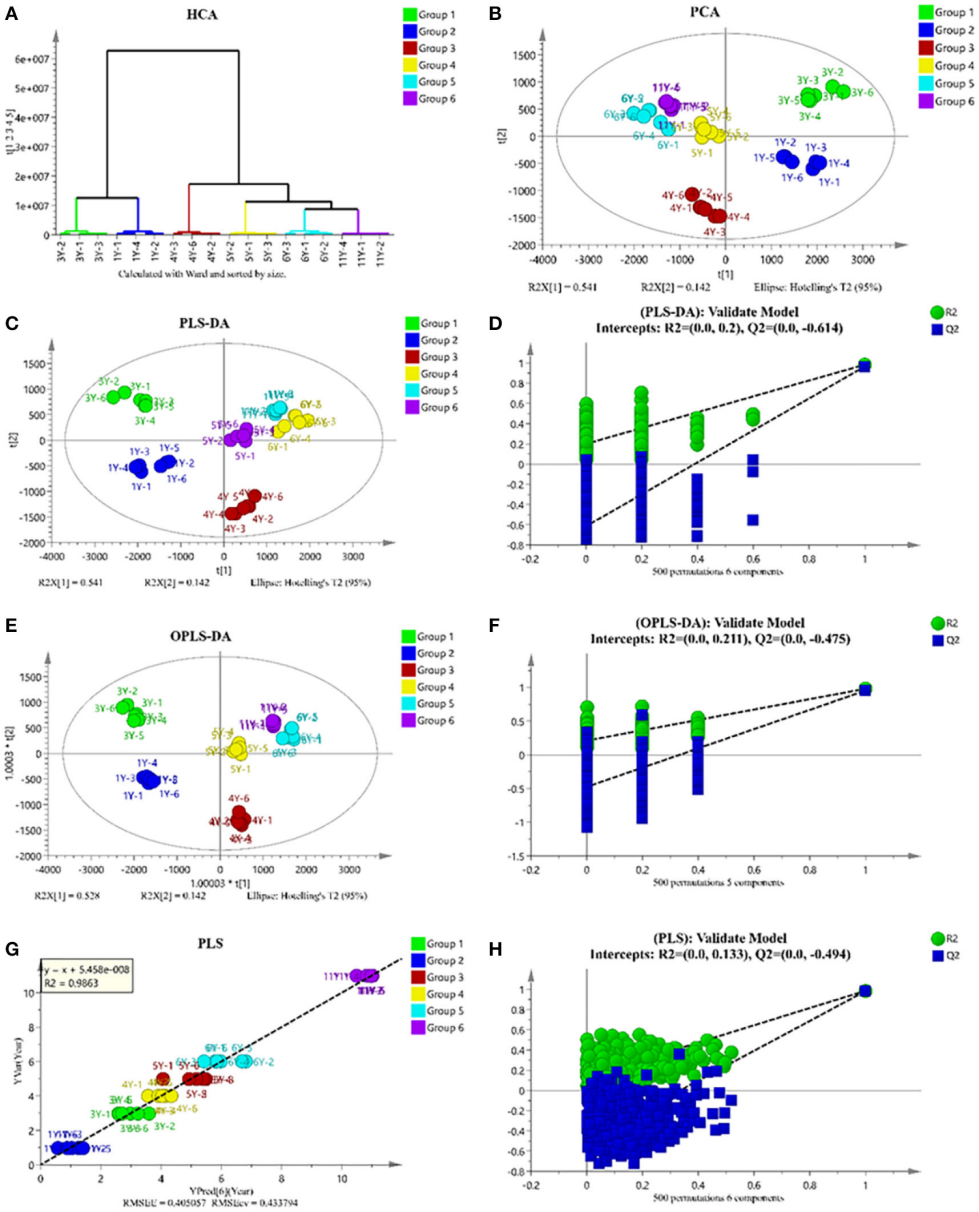
Multivariate analysis of LC-MS-based metabolomics data of CP with different storage years. clustering analysis **(A)**; score plots of principle component analysis **(B)**; partial least squares data analysis (PLS-DA) **(C)**; permutation test result of PLS-DA **(D)**; orthogonal partial least squares data analysis (OPLS-DA) **(E)**; permutation test result of OPLS-DA **(F)**; PLS regression model for prediction of storage years **(G)**; permutation test result of PLS **(H)**.

The original MS data were uploaded to MS-DIAL for processing and then analyzed by SIMCA-P. Although the changes of main PMFs were not regular, cluster analysis divided these samples into two groups: 1Y and 3Y in one group, 4Y, 5Y, 6Y, and 11Y in the other group (Figure 2A). As shown in Figure 2B, the closer the spatial distance between samples is, the more similar the samples are, while the farther the samples are, the greater the differences are. The score chart of principal component analysis also shows the same result. As shown in Figures 2C,E, we established the PLS-DA model (*R*^2^*X* = 91.3%, *R*^2^*Y* = 98.5%, *Q*^2^ = 97.5%) and OPLS-DA model (*R*^2^*X* = 91.3%, *R*^2^*Y* = 98.3%, *Q*^2^ = 96.7%). Cross-validation with 500 displacement tests shows that the model is reliable (Figures 2D,F). To determine the correlation between CP composition and storage time as a continuous Y variable to establish a PLS linear model (Figures 2G,H).

**Figure 3:**
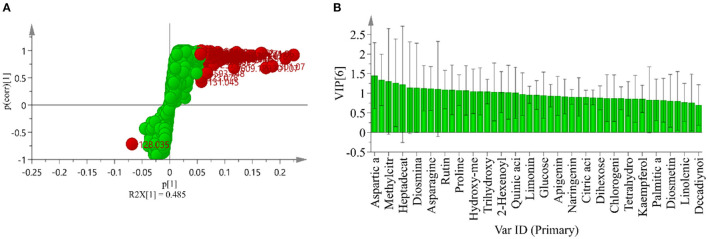
*S*-plot of CP samples under different storage years **(A)** and the order of contribution value of antihyperglycemic and antioxidant activity **(B)**.

To explore the marker compounds contributing to the classification of different years of CP, the *S*-plot profiling was analyzed. As shown in Figure 3A, the many red points indicated the high *p*[1] values, which meant the shifting from the dataset. It also listed the order of the contribution of each marker compound to the biological activities (Figure 3B). They mainly included aspartic acid, methylcitric acid, heptadecatrienoic acid, and diosmin.

### The Identification of Marker Compounds Responsible for the Classification on CP With Different Storage Years

To identify the marker compounds, two approaches were applied in analyzing their chemical structures. Firstly, the standards were used to identify the marker compounds. This approach is the most-trusted method for compounds' identification. Secondly, the mass fragment ions (MS/MS ions) were analyzed, and the fragmentation regulation for the typical compound was found. During the identification, these approaches were used in combination for confirming the structure of the marker compound. All the main marker compounds are described in Table 3, which listed 47 marker compounds discriminating different years of CP. The high-resolution *m/z* and MS/MS results are also presented (Supplementary Figure 1).

**Table 3:** The critical metabolites responsible for distinguishing the storage years of CP.

**No**.	**Var**.	**t_**R**_ (min)**	***m/z***	**MS**	**MS/MS**	**VIP**	**Identification**
1	237	1.27	131.0453	M–H	59.01, 88.04, 114.01	3.65	Asparagine (34, 35)
2	1,122	1.31	341.1067	M–H	59.01, 89.02, 119.03	2.42	Dihexose (34, 35)
3	92	1.31	114.0186	M–H	71.01	2.25	Proline (34, 35)
4	450	1.34	165.0387	M–H	59.01, 75.00, 129.01	2.47	Pentonic acid (34, 35)
5	937	1.34	293.0963	M–H	131.04, 158.97, 203.06	1.77	Asparagine-glucoside (23, 35)
6	569	1.35	191.0552	M–H	59.01, 85.02, 93.03	6.65	Quinic acid (23, 35)
7	642	1.35	203.0652	M–H	70.03, 114.02, 131.04	2.06	Propyl pentoside
8	522	1.35	179.0546	M–H	59.01, 71.01, 89.02	1.98	Glucose (34, 35)
9	242	1.36	132.0289	M–H	59.01, 75.00, 88.03	2.00	Aspartic acid
10	568	1.40	191.0549	M–H	59.01, 85.02	6.33	Citric acid (34, 35)
11	665	1.40	205.0338	M–H	72.99, 81.03, 99.00, 125.02	2.05	Methylcitric acid (35)
12	1,423	8.27	609.1410	M–H	369.06, 399.07, 489.10	1.83	Hesperidin (35, 36)
13	611	9.03	195.1377	M–H	61.98, 92.39, 180.04	1.89	Dihydroferulic acid (34, 35)
14	1,400	9.05	593.1477	M–H	353.06, 383.07, 473.10	3.43	Apigenin-6,8-di-C-glycopyranoside (35, 37)
15	1,312	9.05	473.1052	M–H	61.98, 125.02, 265.14, 363.06	1.70	Blumenol B acetyl-glucoside (35)
16	1,142	9.05	353.0636	M–H	61.98, 85.02, 175.03, 193.04, 235.06	1.59	Chlorogenic acid (23)
17	1,271	9.41	433.1105	M–H	85.02, 193.05, 271.06	1.71	Prunin (35, 37)
18	1,442	9.68	623.1575	M–H	312.06, 383.07, 413.08, 503.11	2.12	Diosmetin di-C-glucoside (32, 35)
19	1,068	10.68	327.0482	M–H	57.02, 85.02, 121.03	2.44	Monohydroxytrimethoxyflavone
20	1,307	10.72	463.1214	M–H	301.07, 395.74	1.77	Quercetin-glucoside
21	1,398	11.92	593.1473	M–H	151.00, 285.03	2.87	Kaempferol-3-rutinoside (35, 38)
22	1,120	12.85	341.0648	M–H	71.01, 89.02, 101.02, 179.05, 260.08	1.87	6,7,8,4′-tetramethoxyflavone (34)
23	869	13.66	271.0596	M–H	81.07, 166.11, 205.04	4.30	Naringenin (35)
24	854	13.97	269.0438	M–H	145.02, 192.15	3.72	Apigenin (35, 39)
25	972	14.33	299.0540	M–H	152.67, 160.09, 216.37, 253.09	1.70	Diosmetin (35)
26	1,458	14.40	651.2611	M–H	59.01, 83.01, 113.02, 185.00, 271.16, 307.18, 428.57	3.48	Tetrahydroxy-dimethoxyflavone HMG-glucoside (35)
27	1,417	14.93	607.1625	M–H	301.07	1.65	Diosmin (35)
28	1,432	15.05	609.1793	M–H	301.07	4.33	Rutin (32, 35, 40)
29	918	18.48	285.0750	M–H	115.00, 135.04, 151.00	4.18	Kaempferol (35)
30	1,308	18.67	469.1316	M–H	121.02, 193.04, 427.19	1.82	Limonin (35)
31	1,510	18.76	726.3781	M–H	696.37	1.51	Gly cerophosphoserines (32)
32	1,076	19.65	329.0278	M–H	57.03, 101.94, 199.09	1.64	Trihydroxydimethoxyflavone (35, 41)
33	909	19.75	284.0305	M–H	60.01, 89.02, 179.05, 359.07, 633.25	1.51	(2E)-octenoylcarnitine
34	1,328	19.93	489.2109	M–H	89.02, 169.08, 236.63, 339.19, 445.03	1.87	Isoorientin 6”-O-acetate (35, 42)
35	1,323	20.10	487.1951	M–H	59.01, 133.06, 383.18	2.58	Ichangin (35, 43)
36	810	20.13	256.0229	M–H	107.04, 213.09	1.86	2-Hexenoylcarnitine
37	882	21.19	277.2148	M–H	91.59, 149.09, 193.08, 265.03	1.71	Linolenic acid
38	1,051	21.32	323.0782	M+46	277.21	3.86	Linoleic acid
39	1,006	21.33	308.0541	M–H	85.02, 137.09, 171.09, 211.13, 265.16	2.82	(2E,5Z,7E)-decatrienoylcarnitine
40	1,106	21.34	338.1029	M–H	308.05, 323.08	8.38	Docosanamide
41	1,042	21.92	322.1061	M–H	266.04, 307.08	1.57	Anandamide
42	805	21.95	255.2306	M–H	96.05	1.57	Palmitic acid (23)
43	844	22.32	265.1459	M–H	96.96	1.80	Heptadecatrienoic acid
44	441	22.56	163.1116	M–H	60.83, 99.71, 116.92	1.51	Decadiynoic acid (35)
45	888	23.15	279.2316	M–H	61.98, 158.75, 210.05	3.50	Octadecadienoic acid (23, 35)
46	382	23.66	152.9945	M–H	106.92	1.74	Non-Adienoic acid
47	1,362	23.81	555.2806	M–H	61.98, 225.00, 402.00	2.63	Cucurbitacin E

The VIP value presented the contribution of each compound, whose content varied significantly during storage. As our previous study in tea storing, fatty acids were susceptible to storage under uncontrolled conditions (23). In Table 3, most of the lipids were tentatively identified by referencing the LIPID MAPS^®^ database. These lipids usually had a long retention time and showed regular regulation of mass fragmentation. Furthermore, some hydroxycinnamic acids were marker compounds, which were supposed to be accumulated by hydrolysis of conjugated phenolic acids during storage. As the main compounds of CP, many flavonol glycosides and PMFs (glycoside) were marker compounds, mainly including rutin, naringenin, kaempferol, tetrahydroxy dimethoxyflavone HMG-glucoside, and apigenin-6,8-di-C-glycopyranoside.

### Antioxidant Activities of Different CP With Different Storage Years

**Figure 4:**
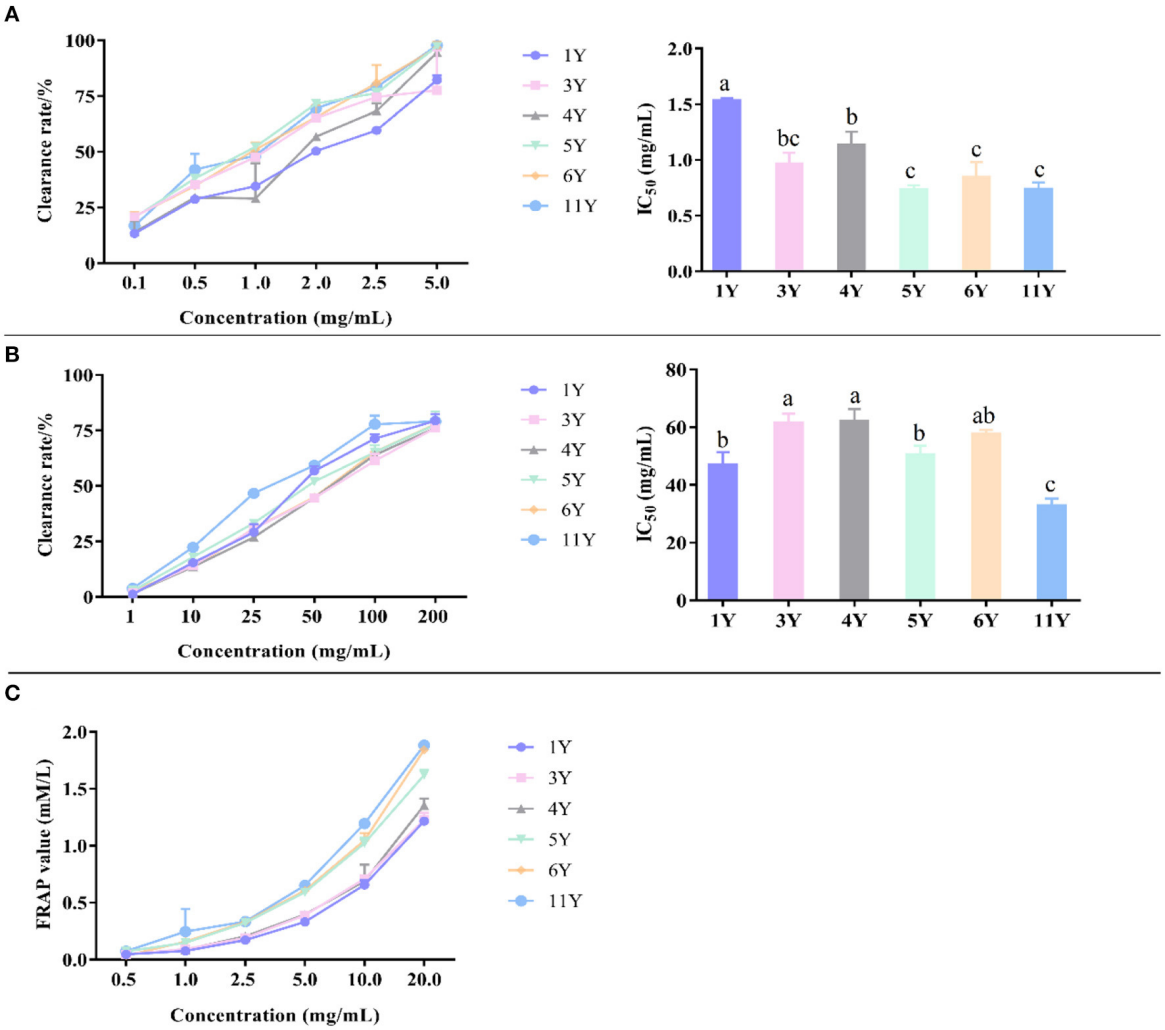
Antioxidant activity of different CP by DPPH **(A)**, ABTS **(B)**, and FRAP **(C)**. Different letters in a column indicate a statistically significant difference (*p* < 0.05), by one-way ANOVA (Tukey' multiply comparisons test).

Each method of determining antioxidant capacity has its limitations and may cause biases (44). Therefore, a single measurement method is not perfect when evaluating antioxidant capacity. In this work, the antioxidant capacity of CP was investigated by combining three methods. It should be noted that the smaller the IC_50_ value, the stronger the scavenging ability and antioxidant capacity. As summarized in Figure 4, different antioxidant activities were observed among the 6 CP samples tested.

The free radical scavenger can capture the single electron of DPPH and make its color lighter so that the maximum absorption wavelength will linearly decrease. The decrease of light absorption value indicates that the antioxidant capacity is enhanced, so as to evaluate the antioxidant capacity of the test samples (45). According to Figure 4A, the DPPH radical scavenging activity of IC_50_ were, in an ascending order, 1.529 ± 0.023 mg/mL (1-year), 1.132 ± 0.123 mg/mL (4-year), 0.963 ± 0.100 mg/mL (3-year), 0.842 ± 0.137 mg/mL (6-year), 0.736 ± 0.062 mg/mL (11-year), and 0.734 ±0.038 mg/mL (5-year), respectively. Thus, CP stored for 5-years displayed the strongest antioxidant capacity, followed by CP stored 11-years, while the weakest antioxidant capacity was detected in CP stored 1-year. In a previous study, the antioxidant capacity of fruits, vegetables, and other plants generally increased after storing for longer at cold temperature (46). Such increase could be related to the increase of antioxidant compounds over time. In this study, the antioxidant active compounds may mainly include polyphenols, fatty acids, organic acids, and amino acids.

ABTS formed ABTS^+^ with hydroperoxide and peroxidase, and the maximum absorption wavelength of the mixture of ABTS^+^ and iron–myoglobin was 820 nm, 734 nm, and 650 nm. The OD value of the mixture at the maximum absorption wavelength decreased in the presence of a hydrogen donor. The antioxidant capacity of antioxidants directly affects the decreased degree of OD value, and so the decreased degree of OD value can reflect the antioxidant capacity (47). According to Figure 4B, the ABTS radical scavenging activity of IC_50_ was, in an ascending order, 61.86 ± 4.41 mg/mL (4-year), 61.36 ± 3.34 mg/mL (3-year), 57.503 ± 1.588 mg/mL (6-year), 50.277 ± 3.301 mg/mL (5-year), 46.763 ± 4.631 mg/mL (1-year), and 32.797 ± 2.405 mg/mL (11-year). Thus, the strongest antioxidant capacity was detected is CP stored for 11-years, followed by the storage time of 1-, 5-, 6-, 3-, and 4-years. These results indicate that the chemical composition changes of CP during storage affect its antioxidant capacity.

The principle of FRAP method is that Fe^3+^-tripyridyltriazine (TPTZ) has the maximum absorption peak at 593 nm, and is reduced to Fe^2+^ form under the action of antioxidant substances, which is blue, and the absorption value increases. The larger FRAP value indicates the stronger reducing ability of antioxidants (48). According to the absorbance of the extract, the corresponding Fe^2+^ concentration can be calculated by the calibration curves and the antioxidant capacity of CP can be obtained. As presented in Figure 4C, the reducing ability of total flavonoids to the ferric ion of CP showed a significant increasing trend with the storage years, indicating that the CP stored for 11-years had the strongest antioxidant capacity.

Owing to the more abundance of flavonoids in CP's, their antioxidant activity is also higher than other fruits or plants (49). In Table 2, as storage time increased, the content of flavonoids increased in general, and therefore the CP (11-year) has the highest antioxidant activity. Furthermore, there might be other compounds in the crude extracts that would have contributed to the activity, such as phenols, organic acids, and so on.

### The Inhibitory Effects of Different Storage Years of CP on α-Amylase and α-Glucosidase Activities

**Figure 5:**
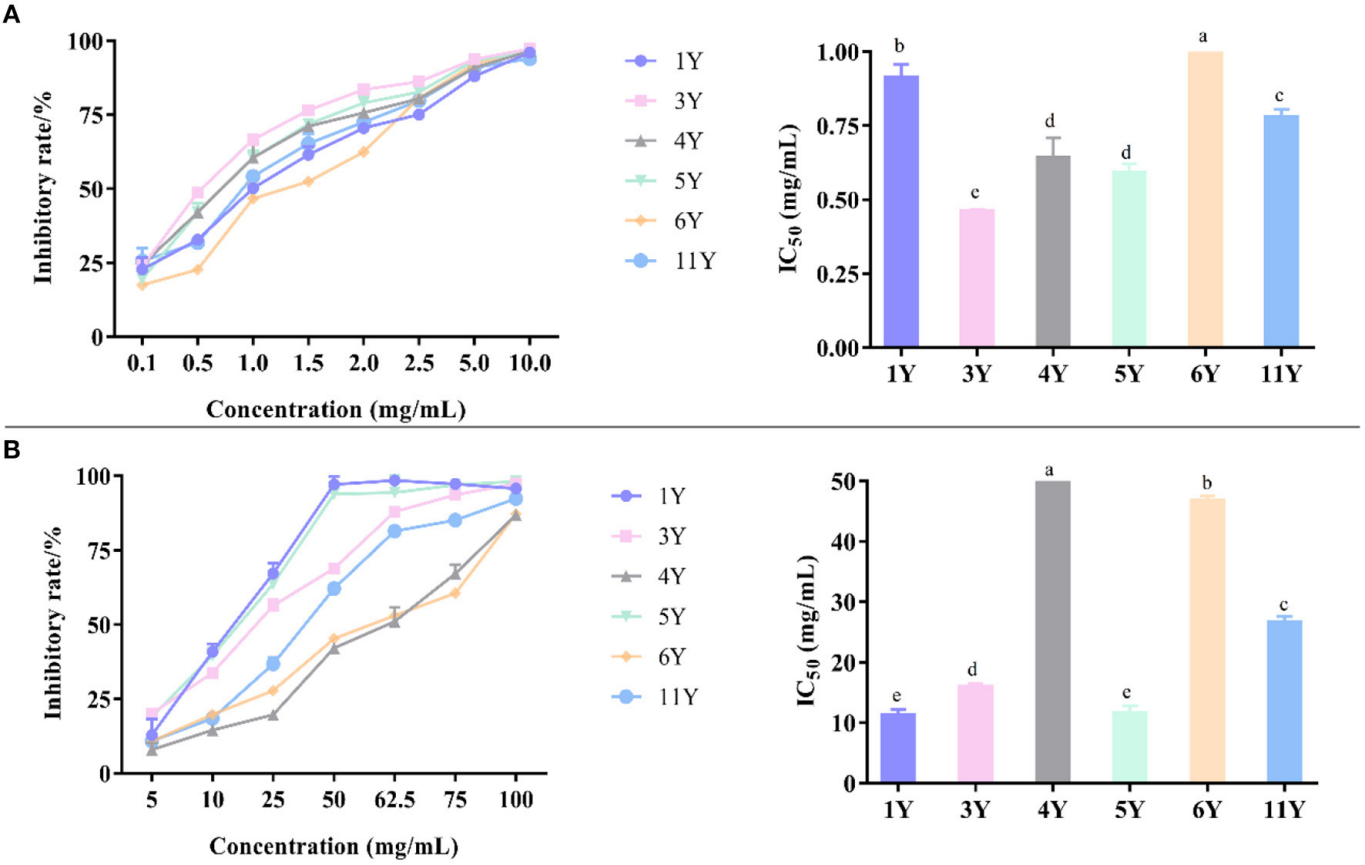
The inhibitory effects of different concentrations of various CP samples and their IC_50_ on α-glucosidase **(A)** and α-amylase **(B)**. Different letters in a column indicate a statistically significant difference (*p* < 0.05), by one-way ANOVA (Tukey's multiply comparisons test).

The experimental principle of α-glucosidase activity was based on the reaction of α-glucosidase with PNPG to produce p-nitrophenol. When the sample solution was added to the system, the activity of α-glucoside enzyme was inhibited in the sample solution, and the amount of *p*-nitrophenol generated at the same time was reduced, which lead to a decrease in the light absorption value (50, 51). According to Figure 5A, α-glucosidase inhibition activity of IC_50_ were, in an ascending order, 1.039 ± 0.022 mg/mL (6-year), 0.911 ± 0.045 mg/mL (1-year), 0.778 ± 0.027 mg/mL (11-year), 0.641 ± 0.069 mg/mL (4-year), 0.591 ± 0.030 mg/mL (5-year), and 0.460 ± 0.007 mg/mL (3-year). Thus, the strongest α-glucosidase inhibition capacity was detected in CP stored for 3-years, followed by the storage time of 5-, 4-, 11-, 1-, and 6-years, while the CP stored for 6-years had the weakest α-glucosidase inhibition capacity.

α-Amylase activity was determined based on the principle of α-amylase cracking starch to produce reducing sugar, which reacted with DNS solution to produce dark compounds. When the sample solution was added to the system, the α-amylase activity was inhibited, and the amount of reducing sugar which lead to the decrease of light absorption at the same time (52, 53). According to Figure 5B, α-amylase inhibition activity of IC_50_ was, in an ascending order, 50.457 ± 1.941 mg/mL (4-year), 46.687 ± 0.831 mg/mL (6-year), 26.653 ± 1.000 mg/mL (11-year), 15.897 ± 0.609 mg/mL (3-year), 11.537 ± 1.289 mg/mL (5-year), and 11.250 ± 0.941 mg/mL (1-year). Thus, the strongest α-amylase inhibition capacity was detected in CP stored 1-year, followed by the storage time of 5-, 3-, 11-, 6-, and 4-years, while the CP stored for 4-years had the weakest α-amylase inhibition capacity.

The analysis showed that there was no definite correlation between the ability of the inhibiting digestive enzyme activity and the different storage years. The antidigestive enzyme ability of CP was also closely related to the growing environment of fruit trees, the way of harvesting and processing, except during its storage years. A Pearson's correlation was analyzed to determine the relationship between marker compound and antioxidant activity/hypoglycemic activities of 6 citrus samples, and the Pearson correlation coefficient is listed in Table 4.

**Table 4:** The Pearson correlation coefficient of marker compounds regarding antioxidant capacity, and inhibitory effects on α-glucosidase/α-amylase.

**Marker compounds**	**Pearson correlation coefficient**				
	**DPPH**	**ABTS**	**FRAP**	**α-glucosidase**	**α-amylase**
Quercetin-glucoside	0.87	0.58	0.67	0.23	0.16
Quinic acid	0.87	0.17	0.86	−0.07	−0.55
Trihydroxydimethoxyflavone	0.86	0.50	0.95	−0.24	−0.19
Hydroxy-methoxy-phenylpropionic acid	0.85	0.09	0.82	−0.01	−0.54
Non-Adienoic acid	0.80	0.03	0.76	0.01	−0.64
Proline	0.78	0.26	0.67	0.00	0.10
Rutin	0.78	0.51	0.97	−0.41	−0.28
2-Hexenoylcarnitine	0.75	0.16	0.69	0.04	−0.63
Naringenin	0.73	0.15	0.64	0.07	−0.64
Propyl pentoside	0.71	0.20	0.60	0.01	– 0.12
Limonin	0.66	0.28	0.84	−0.35	−0.67
Asparagine-glucoside	0.64	0.03	0.52	0.05	−0.46
Asparagine	0.62	0.35	0.59	−0.09	0.29
Decadiynoic acid	0.62	0.01	0.92	−0.55	−0.62
Prunin	0.58	0.22	0.75	−0.29	−0.63
Linolenic acid	0.55	0.26	0.50	0.08	−0.50
Tetrahydroxy-dimethoxyflavone HMG-glucoside	0.48	0.30	0.66	−0.26	−0.43
Citric acid	0.46	0.14	0.70	−0.40	−0.81
Kaempferol	0.46	0.40	0.78	−0.52	−0.47
(2E,5Z,7E)-decatrienoylcarnitine	0.41	0.42	0.22	0.30	−0.25
Kaempferol-glucoside-rhamnose	0.38	0.40	0.65	−0.42	−0.51
Dihexose	0.36	0.23	0.61	−0.40	−0.72
Linoleic acid	0.34	0.44	0.12	0.35	−0.17
Apigenin	0.33	0.39	0.69	−0.54	−0.46
Pentonic acid	0.27	0.28	0.58	−0.48	−0.68
Palmitic acid	0.26	0.45	0.48	−0.31	−0.38
Glucose	0.16	−0.18	0.26	−0.10	−0.82
Blumenol B acetyl-glucoside	0.10	0.47	0.46	−0.51	−0.19
Citrusin	0.10	0.55	−0.24	0.49	0.81
Vicenin-2	0.07	0.48	0.43	−0.50	−0.18
Chlorogenic acid	0.07	0.49	0.45	−0.55	−0.21
Docosanamide	0.06	0.37	−0.29	0.58	0.13
(2E)-octenoylcarnitine	−0.84	−0.40	−0.65	−0.15	−0.11
Isoorientin 6”-O-acetate	−0.73	0.21	−0.70	0.00	0.01
6,7,8,4′-tetramethoxyflavone	−0.59	0.45	−0.54	0.01	0.75
Hesperidin	−0.50	0.00	−0.20	−0.35	−0.38
Methylcitric acid	−0.45	0.08	−0.22	−0.36	−0.61
Ichangin	−0.43	0.32	−0.57	0.33	0.56
Monohydroxytrimethoxyflavone	−0.32	0.34	0.09	−0.57	−0.33
Cucurbitacin E	−0.30	0.35	0.21	−0.81	−0.56
Heptadecatrienoic acid	−0.27	−0.84	−0.25	0.05	−0.01
Octadecadienoic acid	−0.19	−0.09	−0.47	0.56	0.03
Anandamide	−0.18	0.32	−0.45	0.48	0.13
Diosmetin di-C-glucoside	−0.17	0.87	−0.17	0.01	0.57
Diosmetin	−0.13	0.54	−0.06	−0.02	0.52
Diosmin	−0.03	0.62	0.06	−0.17	0.85
Aspartic acid	−0.03	−0.27	−0.57	0.89	0.07

Compounds including quercetin- glucoside, quinic acid, trihydroxydimethoxyflavone, and hydroxy-methoxy-phenylpropionic acid were the marker compounds that had a positive correlation with antioxidant activity. The Pearson correlation coefficient ≥0.5 between compounds, and α-glucosidase/α-amylase showed a potential capability of inhibiting the enzymatic activity.

### Contents of PMFs in Different Fractions of CP

The contents of four flavonoids in different fractions of CP were determined by the established calibration curves. The results showed that hesperidin was rich in the *n*-butanol fraction, and nobiletin, 3,5,6,7,8,3',4'-heptamethoxyflavone, and tangeretin were rich in the dichloromethane fraction, and the content of nobiletin in the dichloromethane fraction was much higher than that in other fractions as shown in Table 5.

**Table 5:** Contents of four kinds of flavonoids in different parts of CP extract.

**Compounds**	**Content (mg/g)**				
	**Petroleum ether**	**Dichloromethane**	**Ethyl acetate**	***n*-Butanol**	**Water**
Hesperidin	0.010 ± 0.001	0.087 ± 0.002	0.263 ± 0.000	0.873 ± 0.008	0.111 ± 0.001
Nobiletin	0.152 ± 0.005	0.730 ± 0.002	0.036 ± 0.000	0.011 ± 0.000	N.D.
3,5,6,7,8,3',4'-Heptamethoxyflavone	0.066 ± 0.001	0.066 ± 0.000	0.013 ± 0.000	N.D.	N.D.
Tangeretin	0.050 ± 0.001	0.067 ± 0.000	0.015 ± 0.000	0.010 ± 0.000	N.D.

*N.D., not detected*.

### The Inhibitory Effects of Different Fractions of CP Extract on α-Amylase and α-Glucosidase

**Figure 6:**
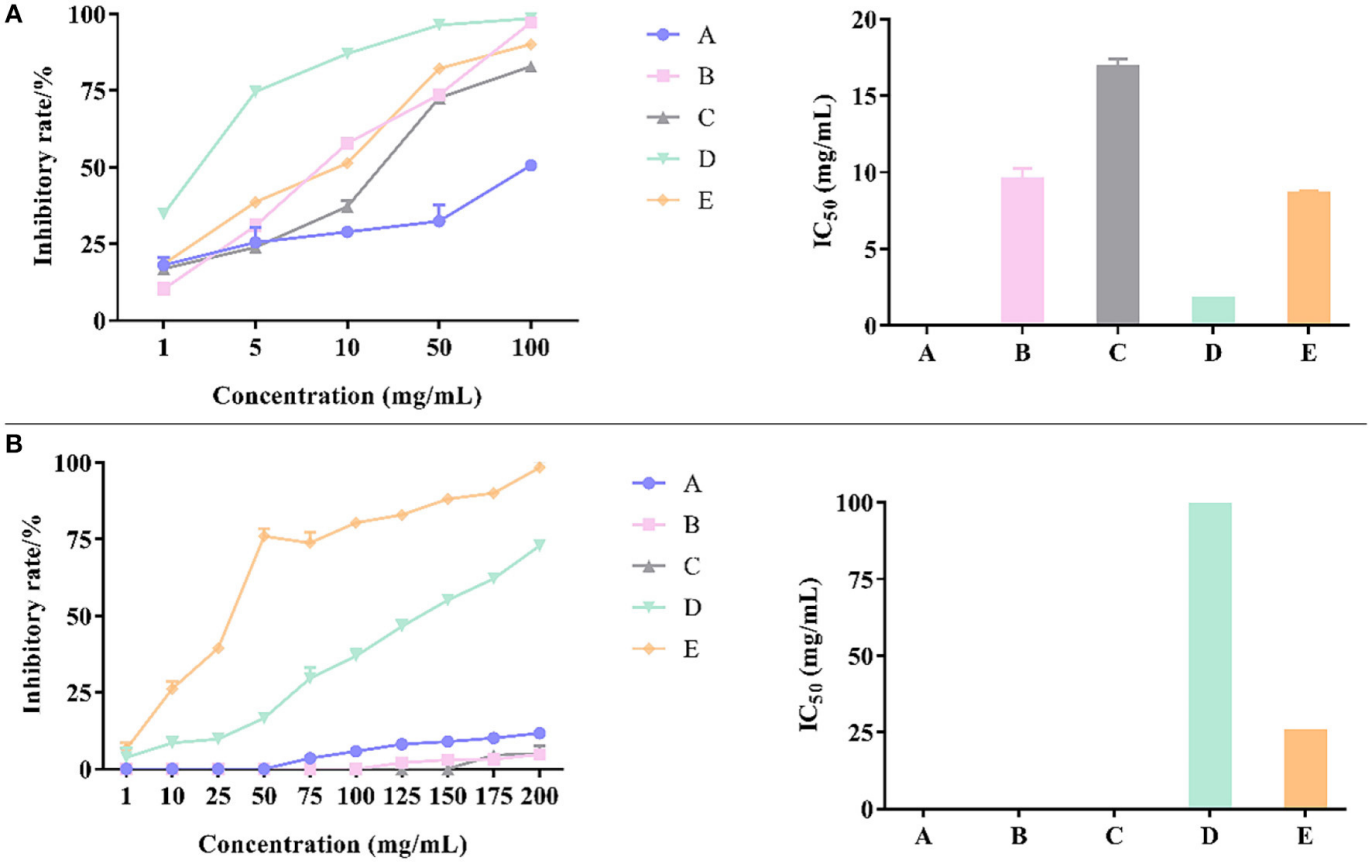
The inhibitory effects of different fractions of CP and their IC_50_ on α-glucosidase **(A)** and α-amylase **(B)**.

Results showed that petroleum ether fraction of CP had no obvious inhibitory activity on α-glucosidase. The capacity of the parts to inhibit the activity of α-glycosidase enzymes can be listed in the order of *n*-butanol > water > dichloromethane > ethyl acetate > petroleum ether. The inhibition rate of *n*-butanol fraction was much higher than other parts under the same concentration as shown in Figure 6A. The IC_50_ value of the *n*-butanol fraction was 1.75 mg/ml, whereas the IC_50_ values of dichloromethane, ethyl acetate, and water fractions were 9.55, 16.84, and 8.61 mg/ml, respectively, which elucidated that there were significant differences in the activity of different fractions.

The petroleum ether, dichloromethane, and ethyl acetate fractions of CP had no obvious inhibitory activity on α-amylase but *n*-butanol and residual extract had significant inhibitory activity on α-amylase. The inhibition rate of *n*-butanol fraction was much higher than other parts under the same concentration as shown in Figure 6B. The IC_50_ value of residual extract was 25.22 mg/ml and that of *n*-butanol fraction was 101.10 mg/mL. Therefore, the inhibition of α-amylase was mainly caused by hydrophilic molecules.

### Antioxidant Activity of Different Fractions of CP

**Figure 7:**
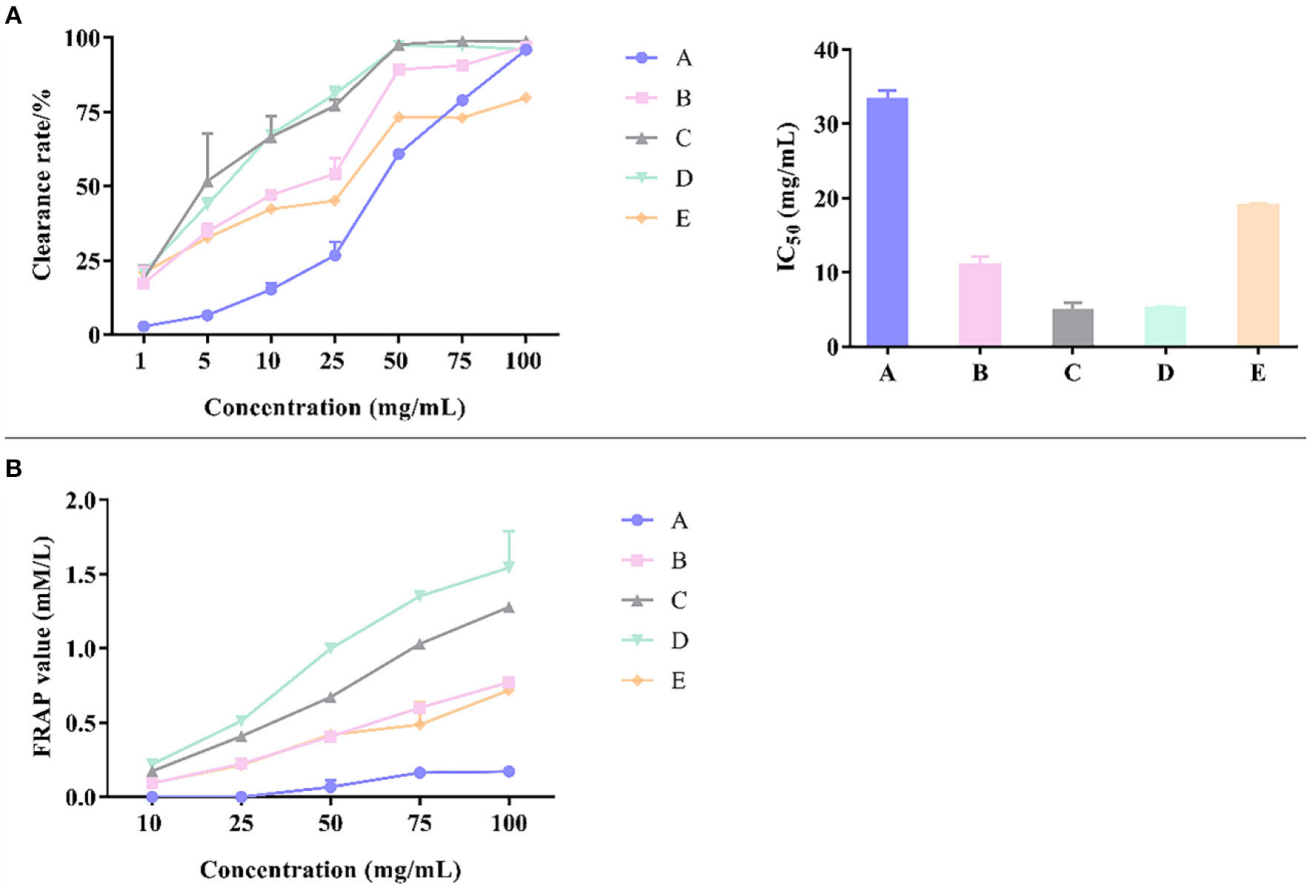
Antioxidant activity of different fractions of CP by DPPH **(A)** and FRAP **(B)**.

According to Figure 7A, the *n*-butanol fraction had the strongest DPPH free-radical scavenging ability and its IC_50_ value was 5.12 mg/mL, while the petroleum ether fraction had the weakest DPPH free radical scavenging ability. The capacity of each fraction to scavenge DPPH radical can be listed in the order of *n*-butanol > ethyl acetate > dichloromethane > water > petroleum ether. Combined with Table 5, it was clear that the antioxidant activities were supposed to be ascribed to hesperidin.

ABTS free-radical scavenging experiment showed that all the fractions had no ABTS antioxidant capacity. The concentration of each fraction was 200 mg/mL, and the content of flavonoids in each fraction was low. Combined with the experimental results in 3.4, the IC_50_ of ABTS scavenging ability of CP of different years was 32.80–61.87 mg /mL, and another evidence listed in Table 2 indicated that the concentration threshold of flavonoid to scavenge ABTS free radical should be much higher than the concentration of flavonoid in the different fractions listed in Table 5.

The reduction ability of Fe^2+^ of different fractions was different, as shown in Figure 7B, which can be listed in the order of *n*-butanol > ethyl acetate > dichloromethane > water > petroleum ether. The reduction ability of petroleum ether fraction was almost 0. This order showed the same tendency with that of DPPH free radical scavenging ability, which was also strongly linked with the contents of flavonoid compounds distributed in different fractions.

Interestingly, in spite of adopting three different antioxidant bioassay platforms, the *n*-butanol fraction was consistently observed to be the most antioxidant agent among all 5 fractions (petroleum ether, dichloromethane, ethyl acetate, *n*-butanol, and water fractions) tested. Flavonoids contents are prolific in CP and are known for having an antioxidant effect. In this study, we only discussed the trend of 4 PMFs (hesperidin, nobiletin, 3,5,6,7,8,3',4'-heptamethoxyflavone, and tangeretin) with the extension of time. Nevertheless, there might be other compounds in the crude extracts that would have contributed to the activity. Therefore, in Table 6, we mainly identified 11 compounds in dichloromethane fraction and 14 compounds in *n*-butanol fraction. Thereinto, vanillic acid, ferulic acid, and other compounds in CP might be good for the antioxidation activity.

**Table 6:** The main compounds identification in dichloromethane and *n*-butanol fractions of CP.

**NO**	**t_**R**_ (min)**	**[M+H]**	**[M–H]**	**λ_max_ (nm)**	**+MS^**2**^**	**–MS^**2**^**	**Identification**
Dichloromethane fraction							
1–1	3.48	295	293	300	211.07, 259.09	131.04, 203.06	–
1–2	3.48	381	379	300	144.10	89.02, 119.03, 179.05, 343.11	Butyl HMG-hexoside (34, 35)
2	4.76	144	142	220, 290, 325	58.06, 70.06, 84.08, 113.02	59.01, 85.02	–
3	9.26	169	167	240	93.03, 125.05	108.02, 152.01	Vanillic acid (35)
4	13.84	195	193	240, 325	89.03, 117.03, 145.02, 177.05	134.03, 178.02	Ferulic acid (35)
5	15.58	611	609	230, 285, 330	303.08	301.70	Hesperidin (35, 36)
6	17.48	193	191	230, 290, 330	57.03, 68.99, 95.08, 119.08	N.D.	Gelseminic acid (34)
7	18.54	728	726	240, 310	N.D.	696.36	Gly cerophosphoserines (32)
8	23.80	373		330	–	–	–
9	24.85	403	–	250, 275, 330	373.09	N.D.	Nobiletin
10	25.49	433	–	330	403.10	N.D.	3,5,6,7,8,3',4'-heptamethoxyflavone
11	26.95	373	–	330	343.08	N.D.	Tangeretin
***n*** **-Butanol fraction**							
1	4.12	342	340	295	306.11	N.D.	–
2	4.55	219	195	290, 310	–	–	Dihydroferulic acid (35)
3	4.76	287	285	225, 275, 330	144.10	105.01	Kaempferol (35, 54)
4	5.48	595	593	275, 330	457.11, 577.15	353.06, 383.07, 473.10	Apigenin-6,8-di-C-glycopyranoside (35, 36)
5	5.73	409	385	245, 330	–	179.06	Roseoside (35, 55)
6	6.49	625	623	225, 280, 330	325.07, 355.08, 409.08	312.06, 383.07, 413.08, 503.11	Chrysoeriol 6,8-di-C-glucoside (35–37)
7	8.07	625	623	235, 325	57.03, 305.88, 367.08, 409.09, 427.10	327.05, 383.07, 413.08, 503.11	Diosmetin di-C-glucoside (35–37)
8	11.11	595	593	250, 340	287.05, 449.10	285.04	Kaempferol-3-rutinoside (35–37)
9	12.83	581	579	240, 325	85.02, 273.07	271.06	Naringenin-7-hesperidoside (32, 35, 56)
10	13.43	463	461	280, 330	313.07, 343.08, 367.08, 427.10	298.04, 341.06	Diosmetin C-glucoside (32)
11	15.59	611	609	280	303.08	301.07	hesperidin
12	24.88	403	–	275, 330	373.09	N.D.	nobiletin
13	25.49	433	–	225, 335	403.10	N.D.	3,5,6,7,8,3',4'-heptamethoxyflavone
14	26.95	373	–	330	343.08	N.D.	Tangeretin

### Identification of Bioactive Compounds in *n*-Butanol and Dichloromethane Fractions by HPLC-DAD-Q-TOF-MS^n^

**Figure 8:**
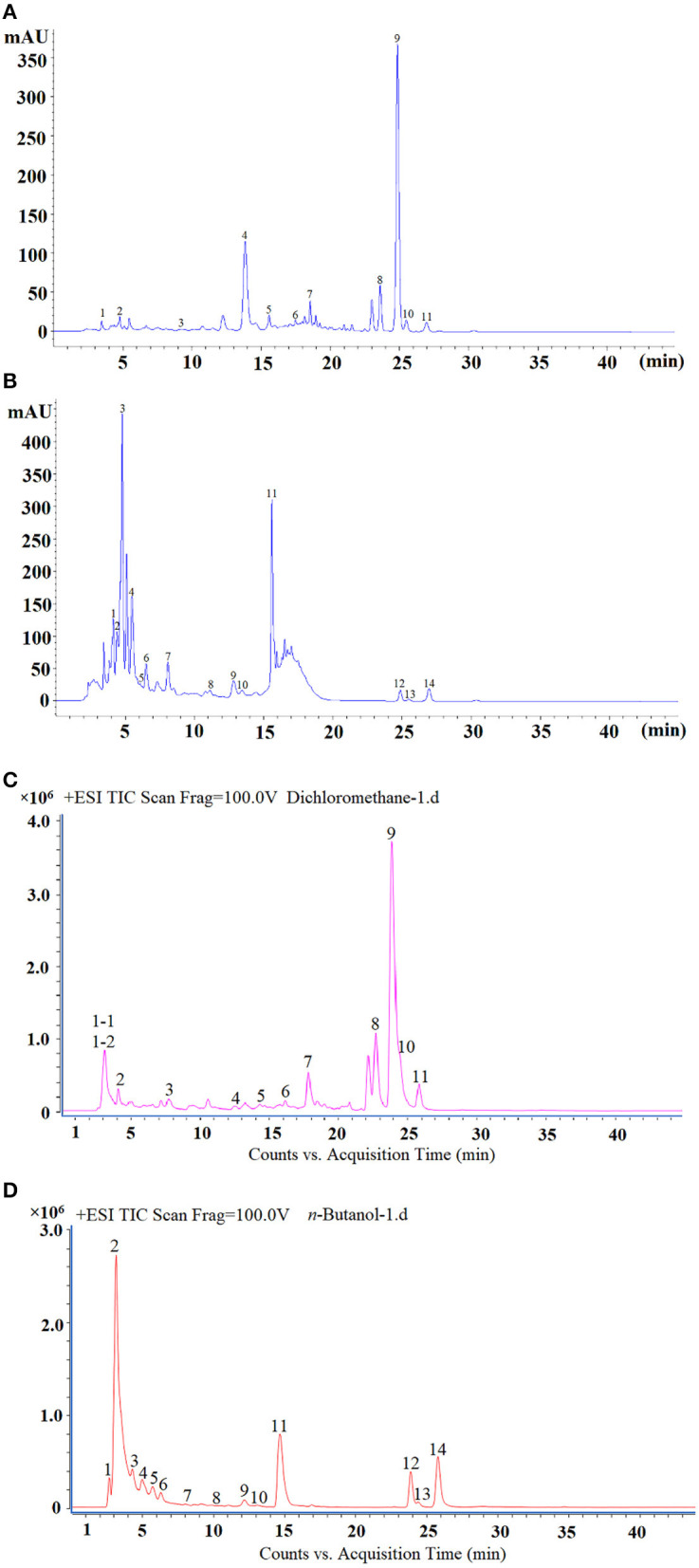
The HPLC spectrum of dichloromethane **(A)**, *n*-butanol fraction **(B)**, total ion chromatogram (TIC) of dichloromethane **(C)**, and *n*-butanol fraction **(D)**.

Since the content of dichloromethane and the activity of *n*-butanol were both higher than other fractions, the two fractions were used for LC-MS/MS analysis and structural analysis. The results are shown in Table 6 and the chromatographic spectrums of the two fractions are shown in Figure 8. The dichloromethane fraction mainly contained ferulic acid, nobiletin, and 3,5,6,7,8,3',4'-heptamethoxyflavone. The *n*-butanol fraction mainly contained kaempferol and hesperidin.

## Conclusion

In this work, a method combining metabolomics analysis, antioxidant capacities, and digestive enzymes inhibition experiment was proposed to evaluate the quality of CP of different storage years and identify potentially active compounds. Untargeted metabolomics analysis revealed significant differences among CP with different storage years, and a total of 47 different compounds were identified from CP, mainly including flavonoids, hydroxycinnamic acids, amino acids, and fatty acids. However, the quantitative analysis of CP with different storage years did not show certain rules. Through this study, we can understand the complex internal mechanism that affects the quality of CP, and further research is needed to reveal the exact mechanism.

## Data Availability Statement

The original contributions presented in the study are included in the article/Supplementary Materials, further inquiries can be directed to the corresponding author/s.

## Author Contributions

MY contributed to conceptualization, methodology, formal analysis, investigation, writing, reviewing, and editing. ZJ contributed to investigation, methodology, and formal analysis. MW contributed to methodology, writing, reviewing, and editing. ZW and LZ contributed to conceptualization, writing, reviewing, editing, project administration, and funding acquisition. MZ and WX contributed to writing, reviewing, and editing. All authors contributed to the article and approved the submitted version.

## Funding

This work was financially supported by the Open Project of Key Laboratory of Modern Preparation of TCM, Ministry of Education, Jiangxi University of Chinese Medicine (Grant No. TCM-202004).

## Conflict of Interest

The authors declare that the research was conducted in the absence of any commercial or financial relationships that could be construed as a potential conflict of interest.

## Publisher's Note

All claims expressed in this article are solely those of the authors and do not necessarily represent those of their affiliated organizations, or those of the publisher, the editors and the reviewers. Any product that may be evaluated in this article, or claim that may be made by its manufacturer, is not guaranteed or endorsed by the publisher.
