# Supplementary material for: Chemical Variation of Chenpi (Citrus Peels) and Corresponding Correlated Bioactive Compounds by LC-MS Metabolomics and Multibioassay Analysis
Source: Front Nutr. 2022 Feb 23;9:825381. doi: 10.3389/fnut.2022.825381 (PMC8905505; doi:10.3389/fnut.2022.825381)

**Supplementary Figure 1** General fragmentation behaviors of marker compounds: MS/MS spectrum of the critical metabolites responsible for distinguishing the storage years of CP.

1


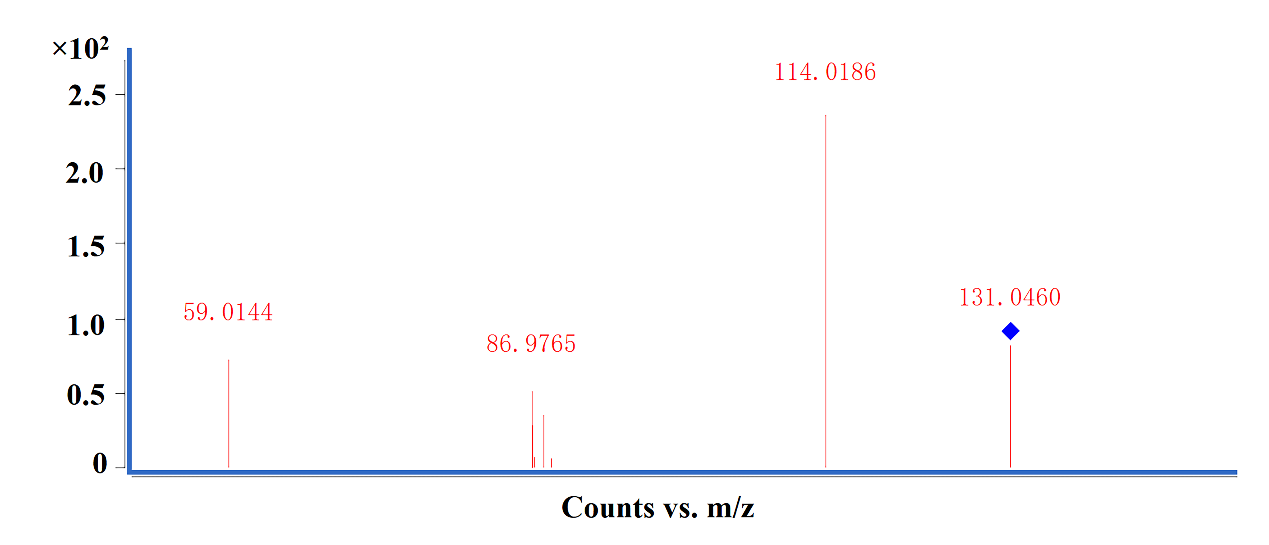


2


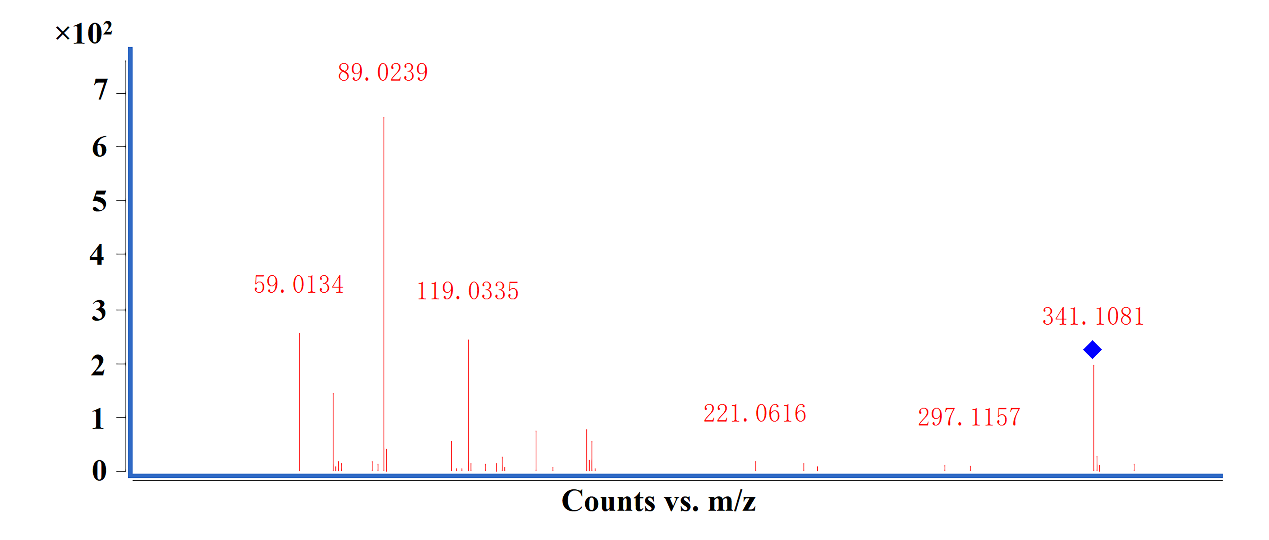


3


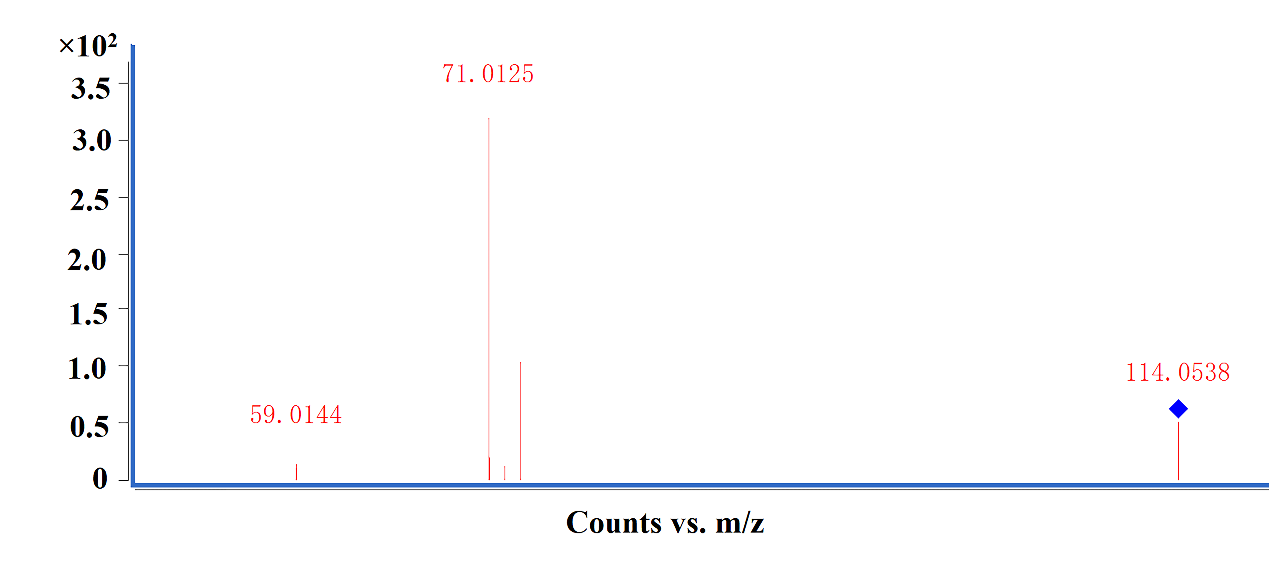


4


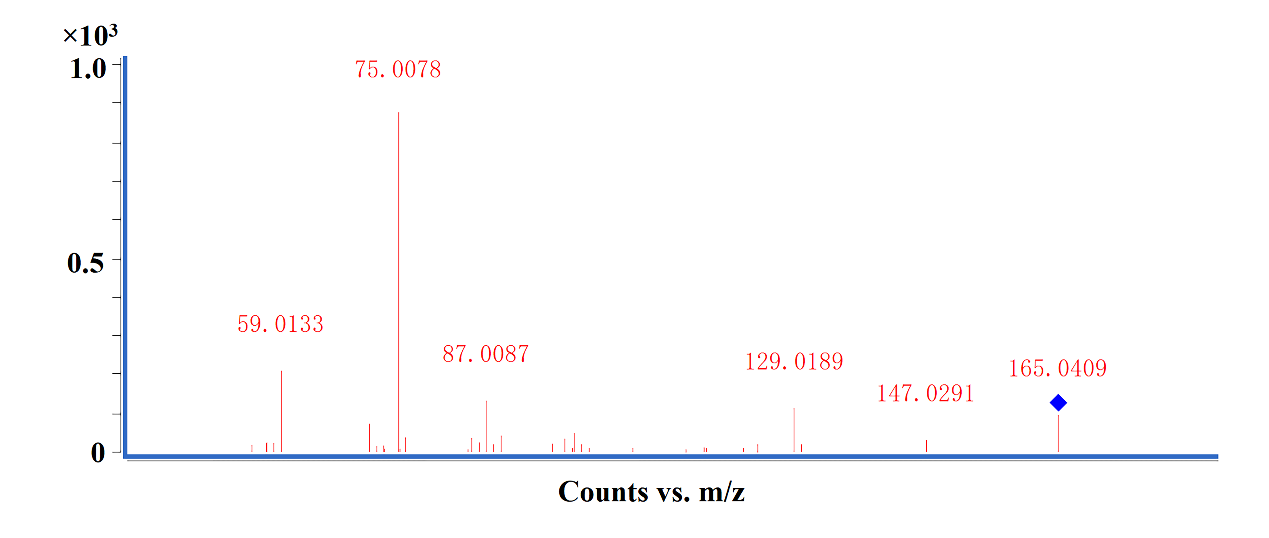


5


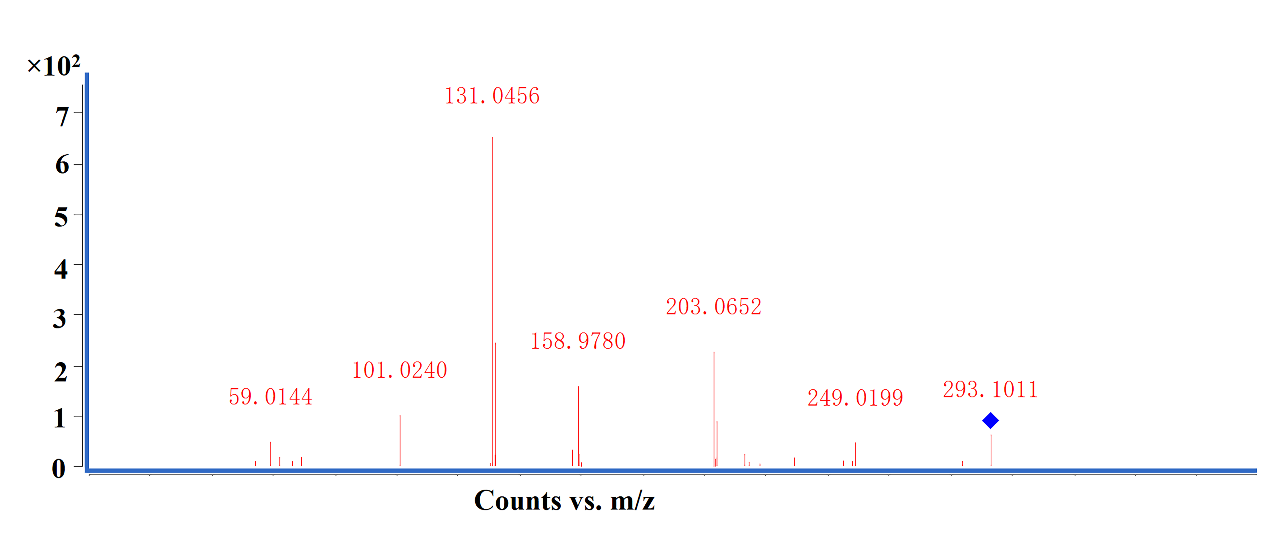


6


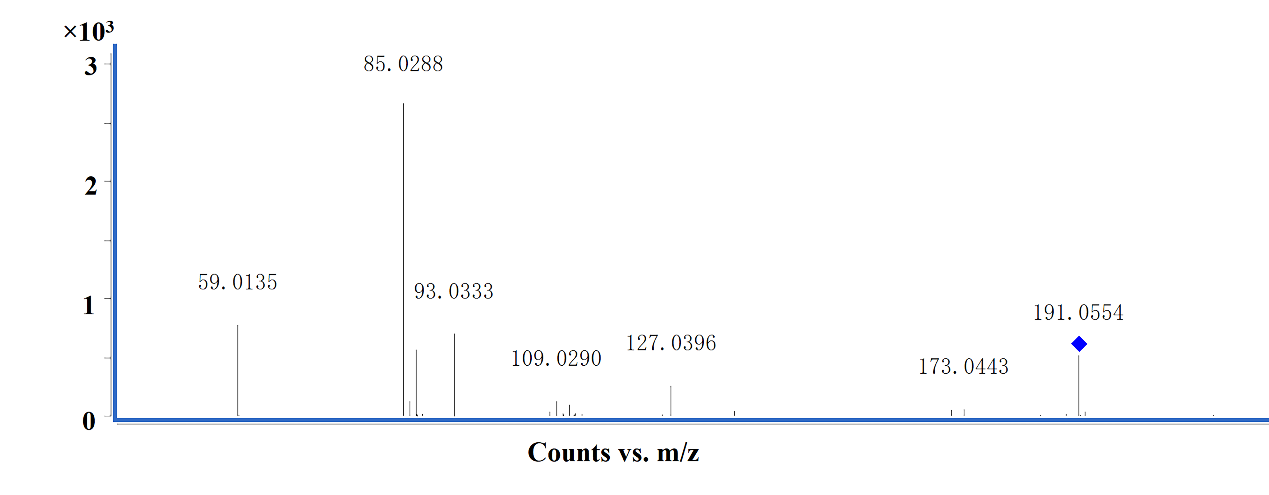


7


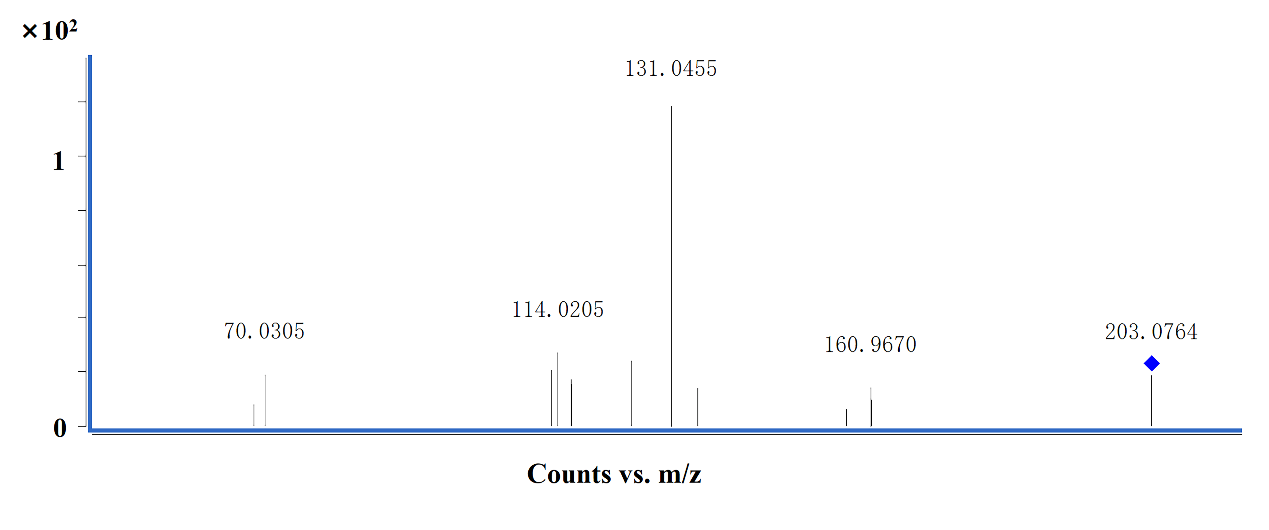


8


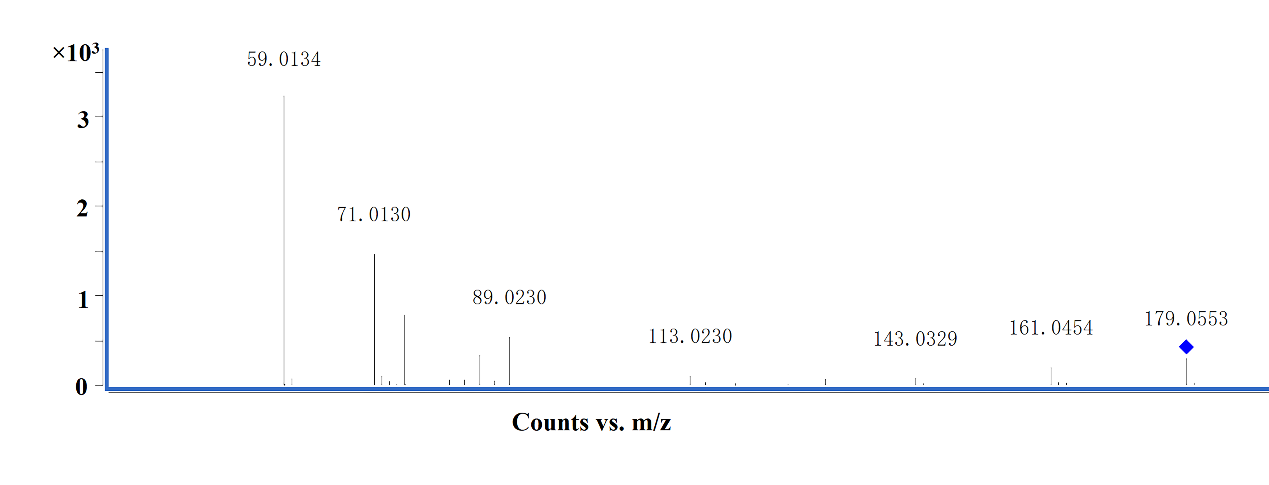


9


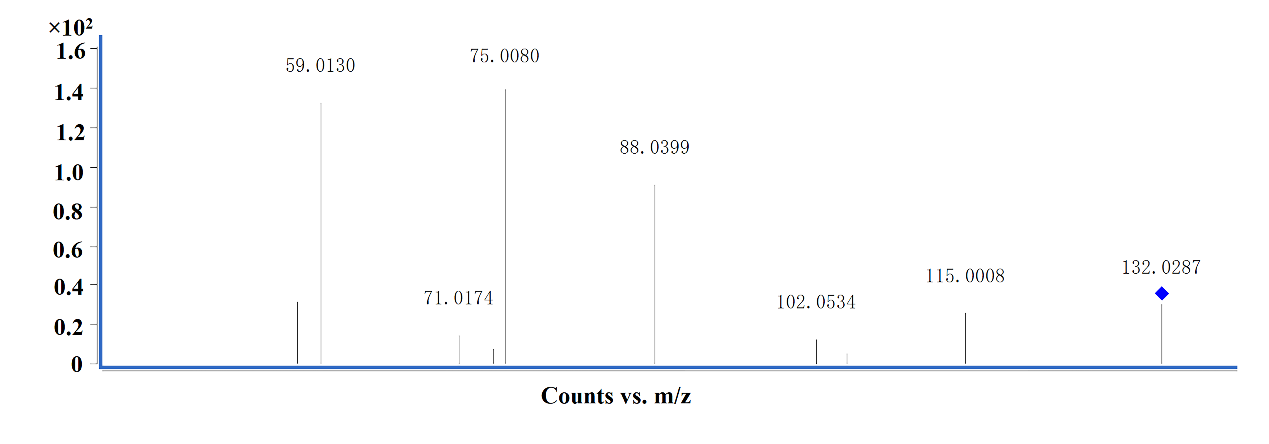


10


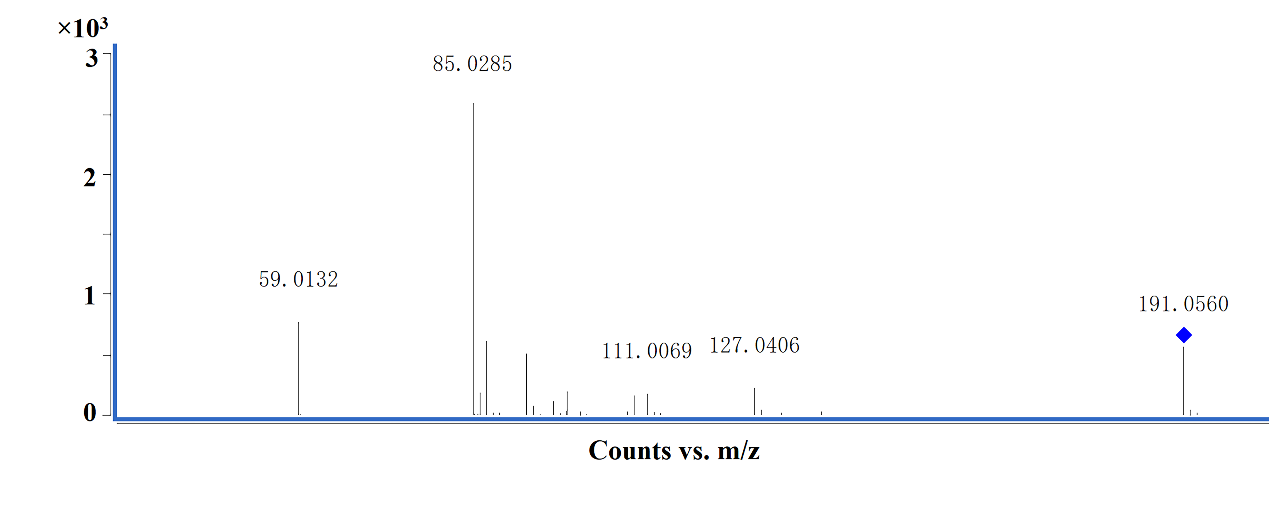


11


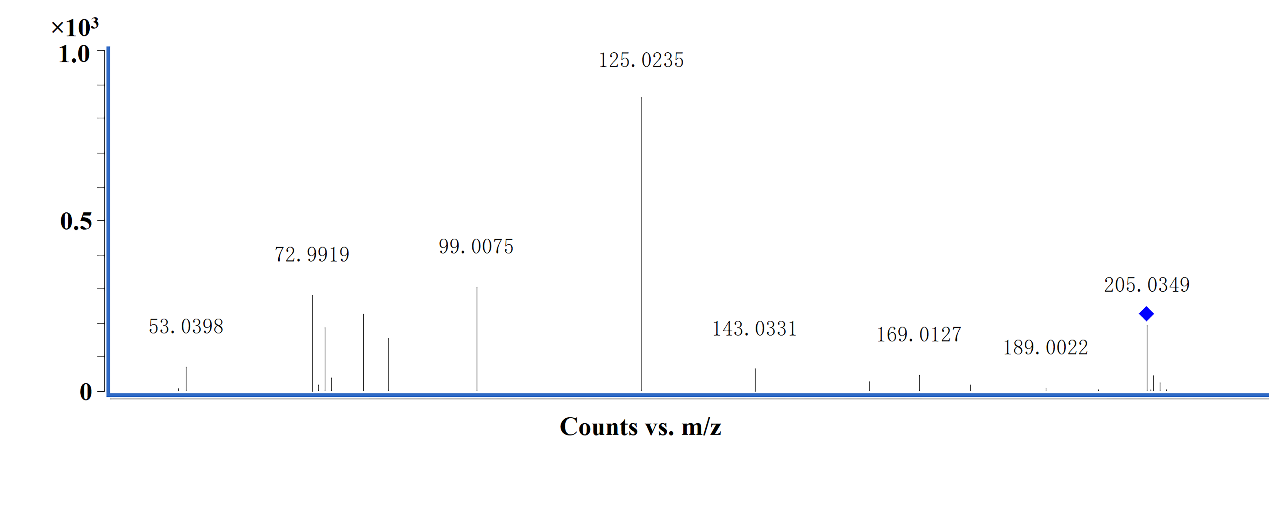


12


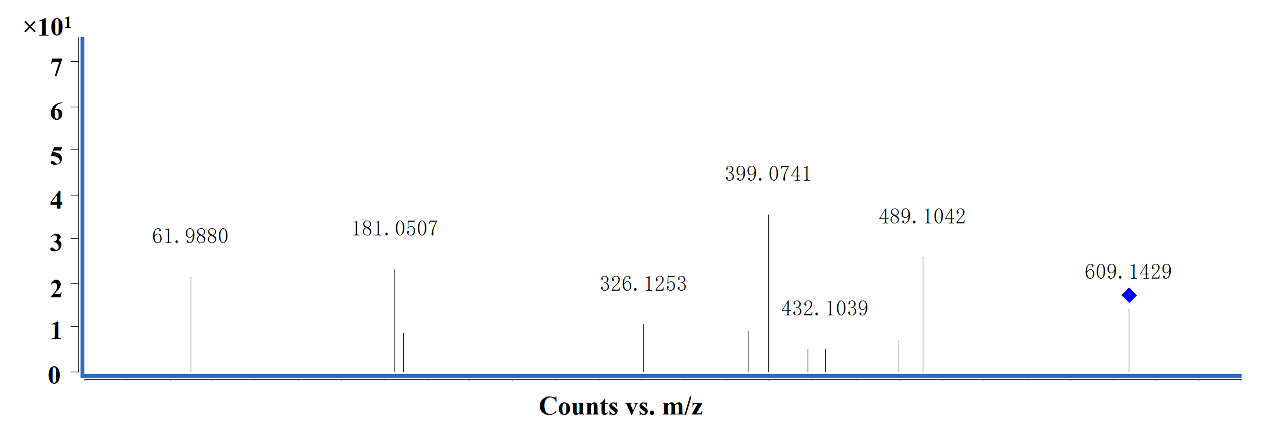


13


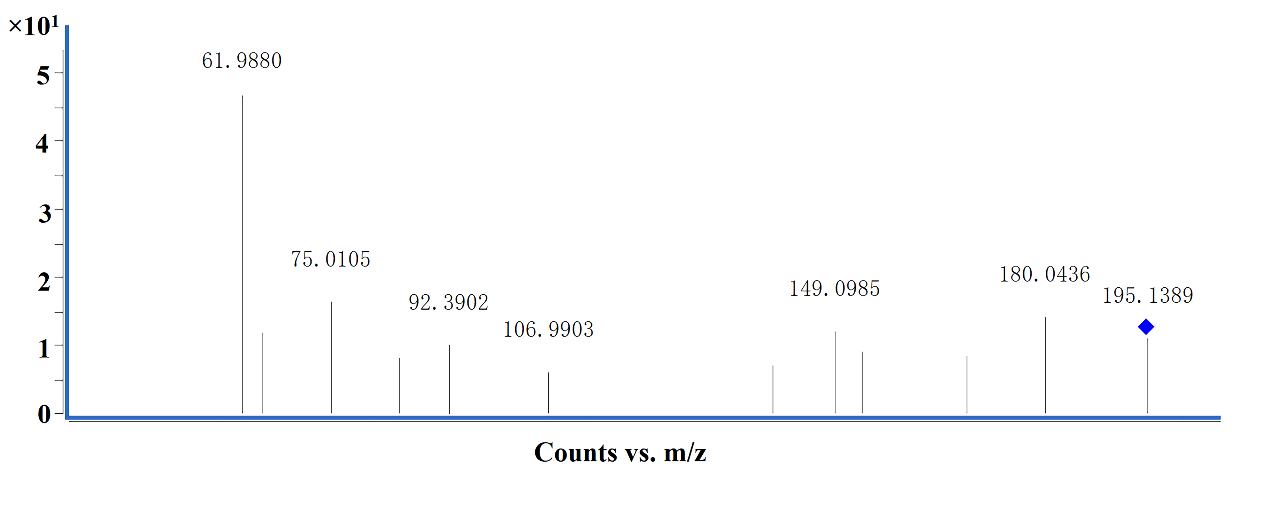


14


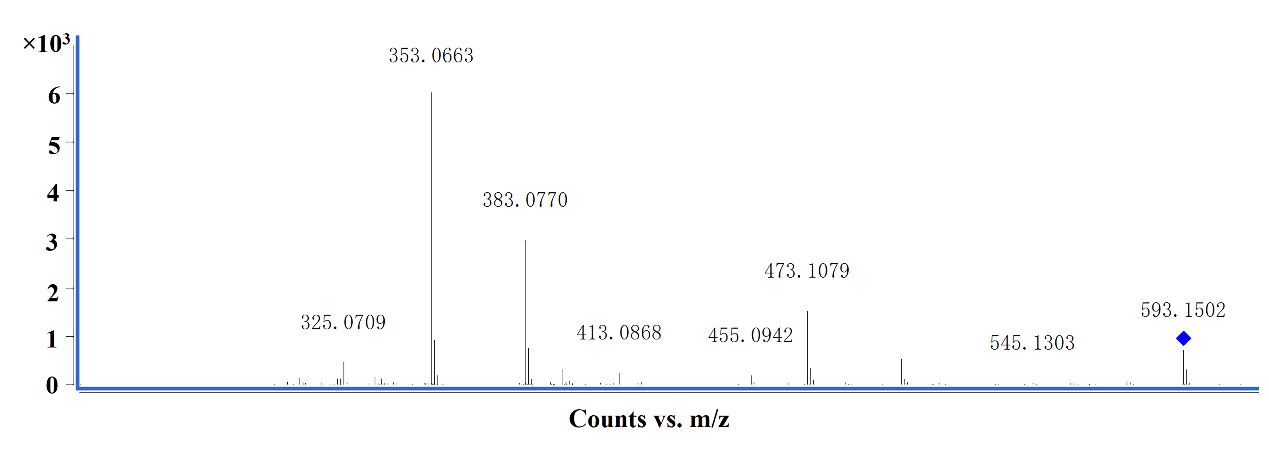


15


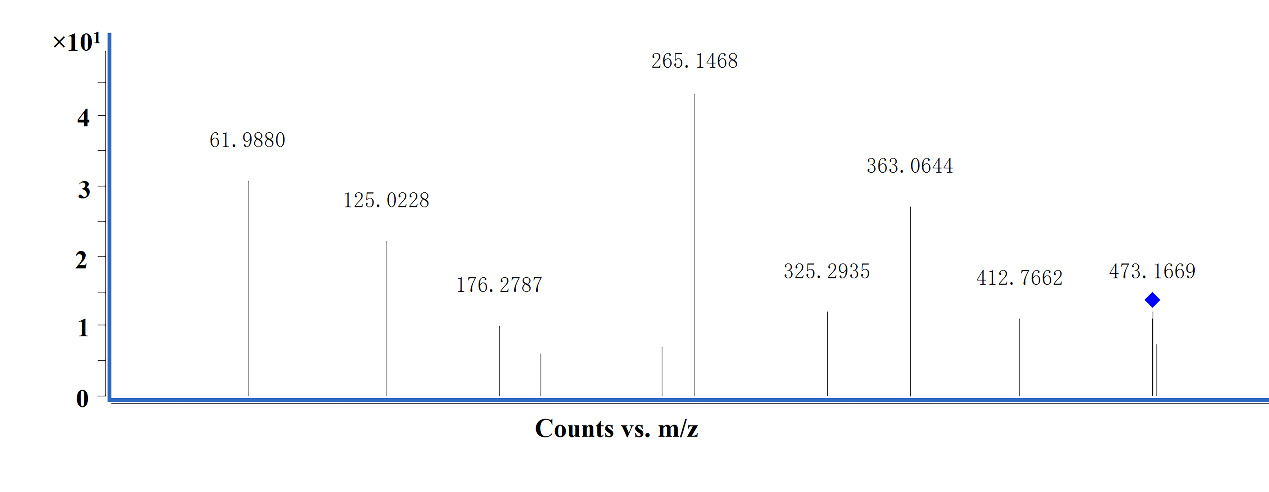


16


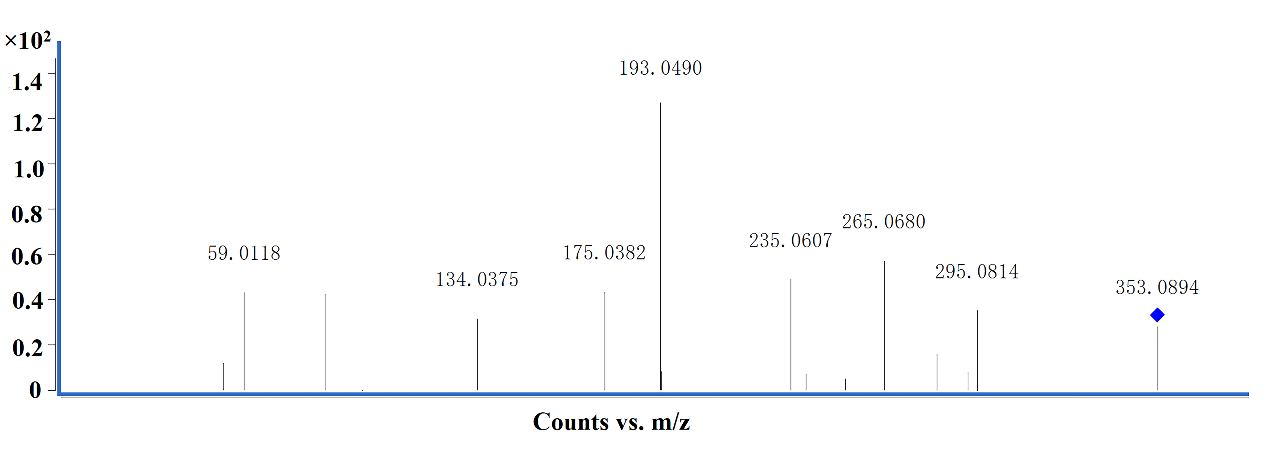


17


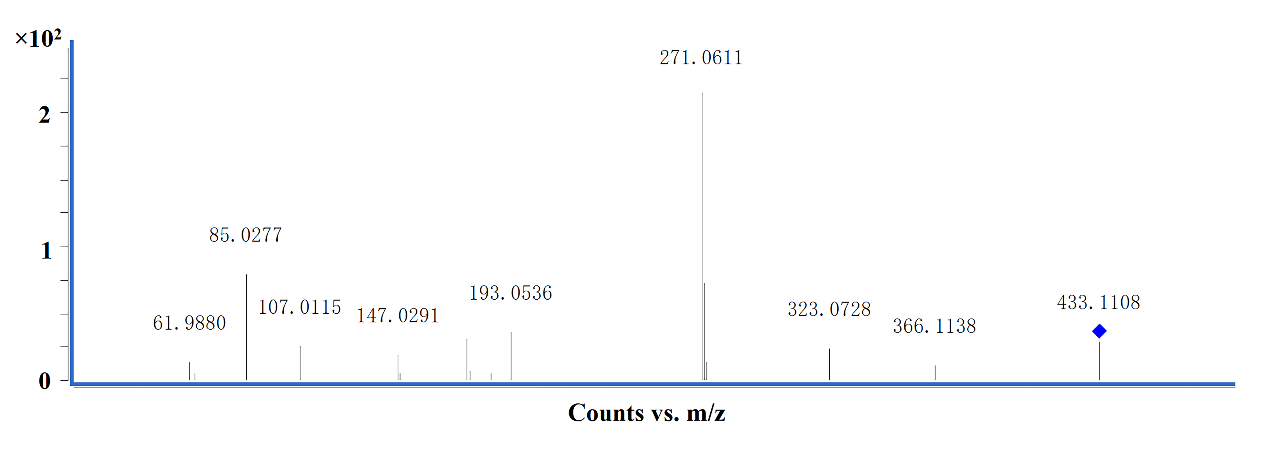


18


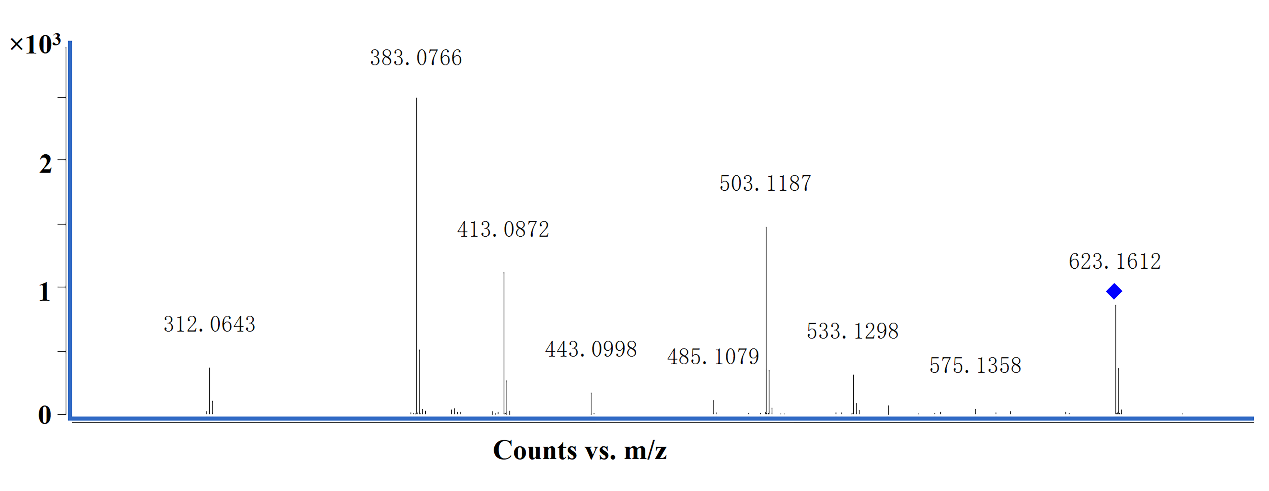


19


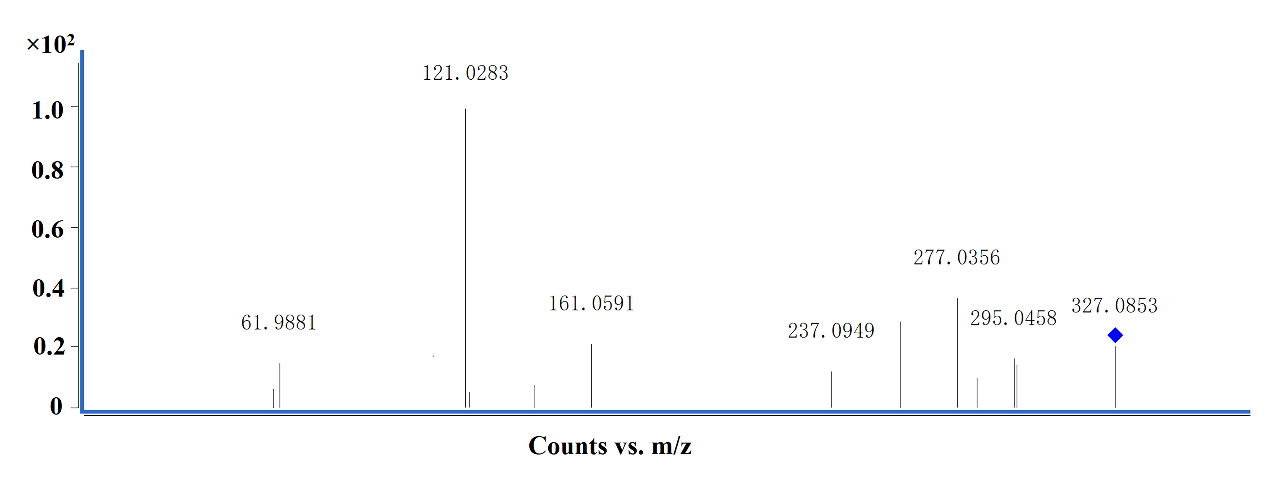


20


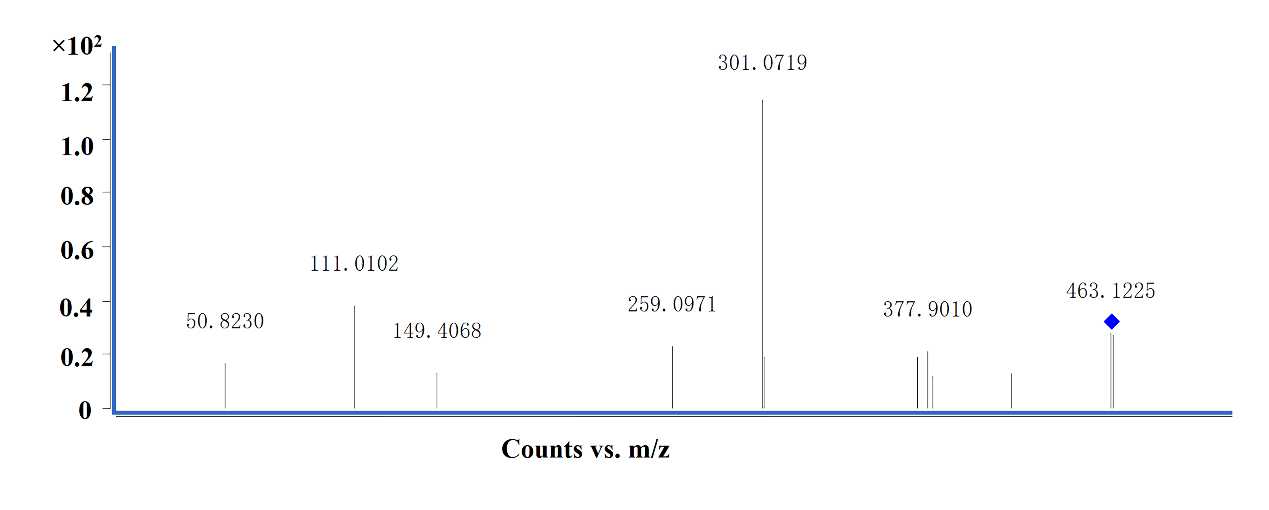


21


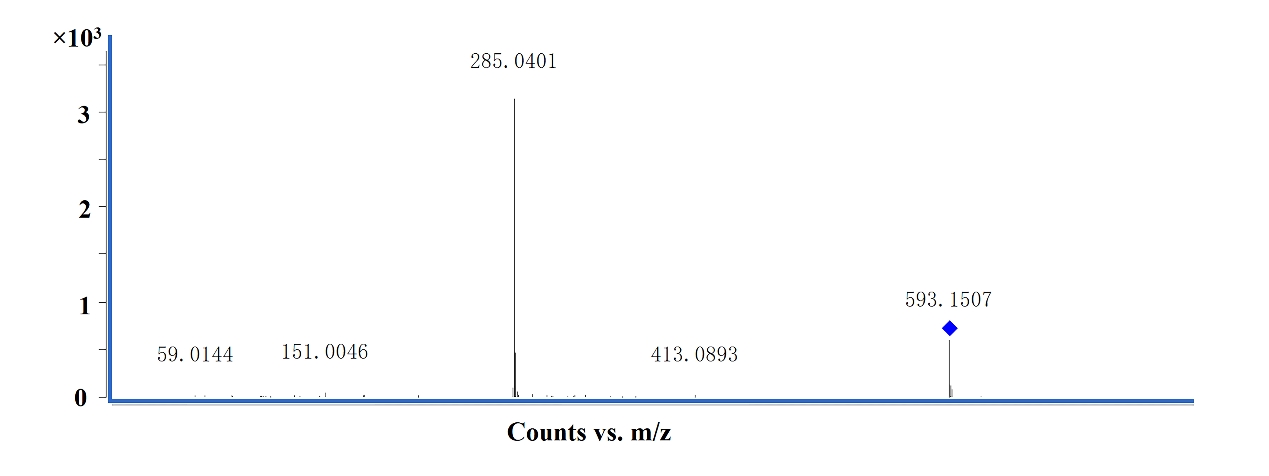


22


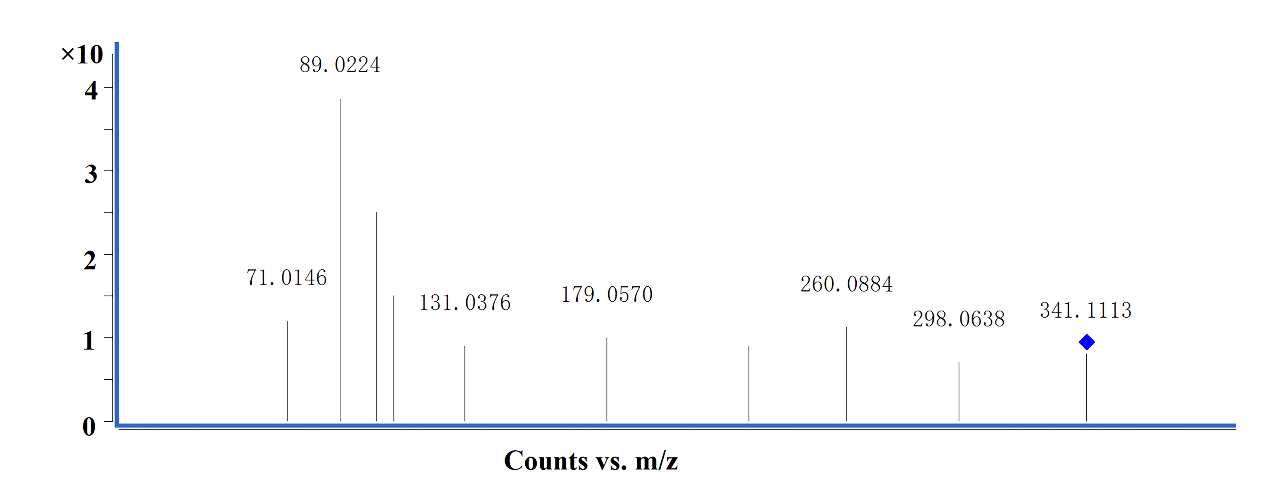


23


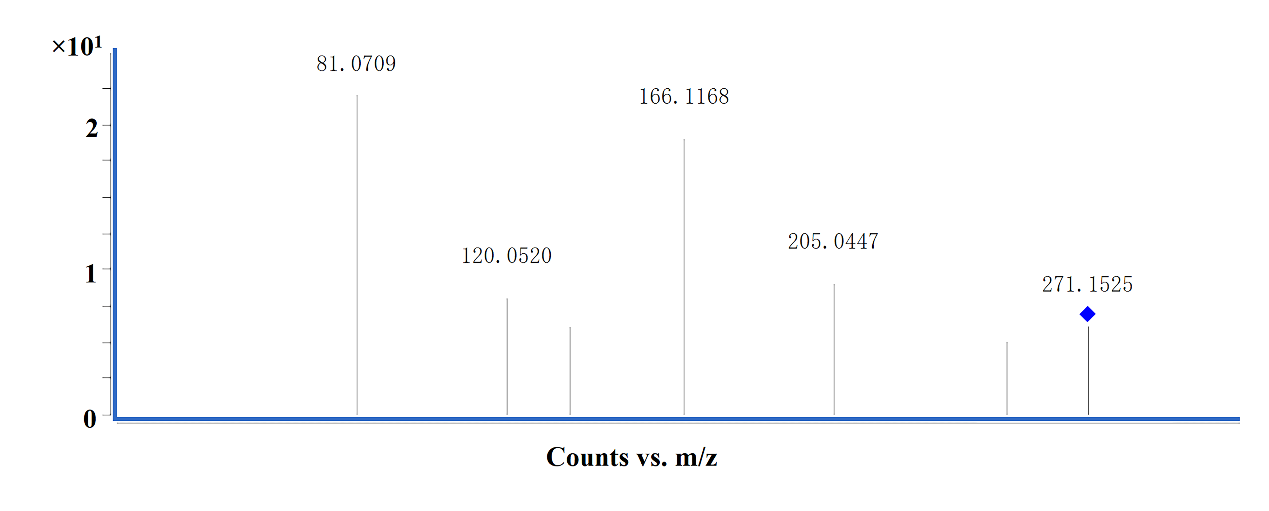


24


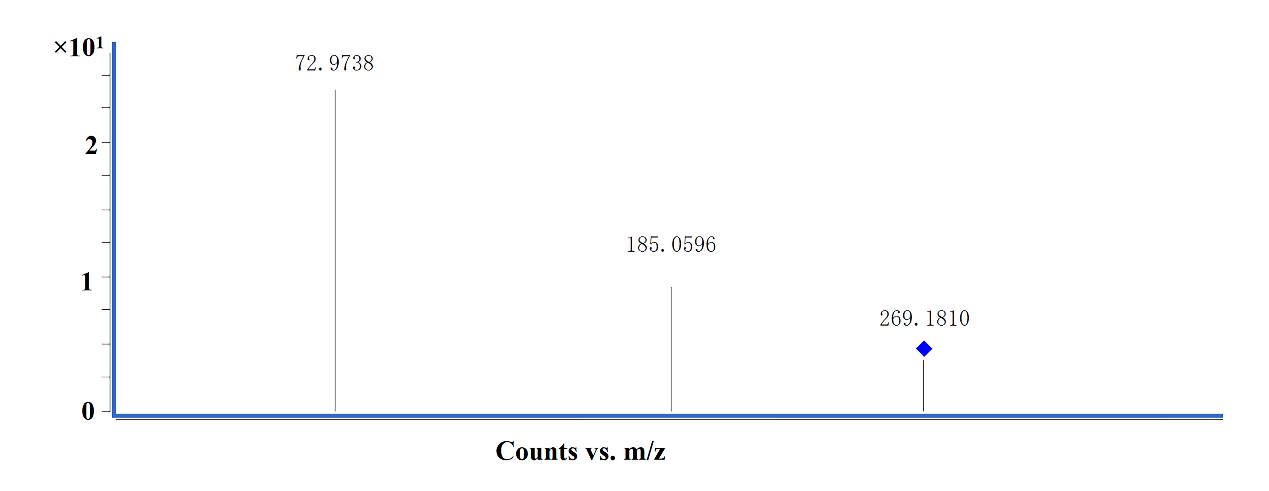


25


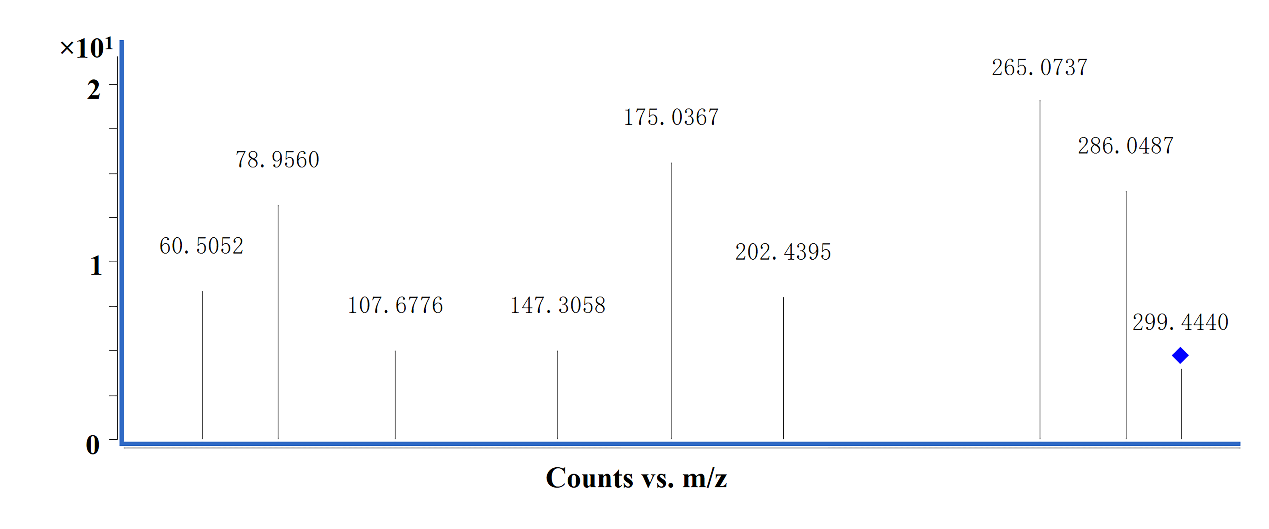


26


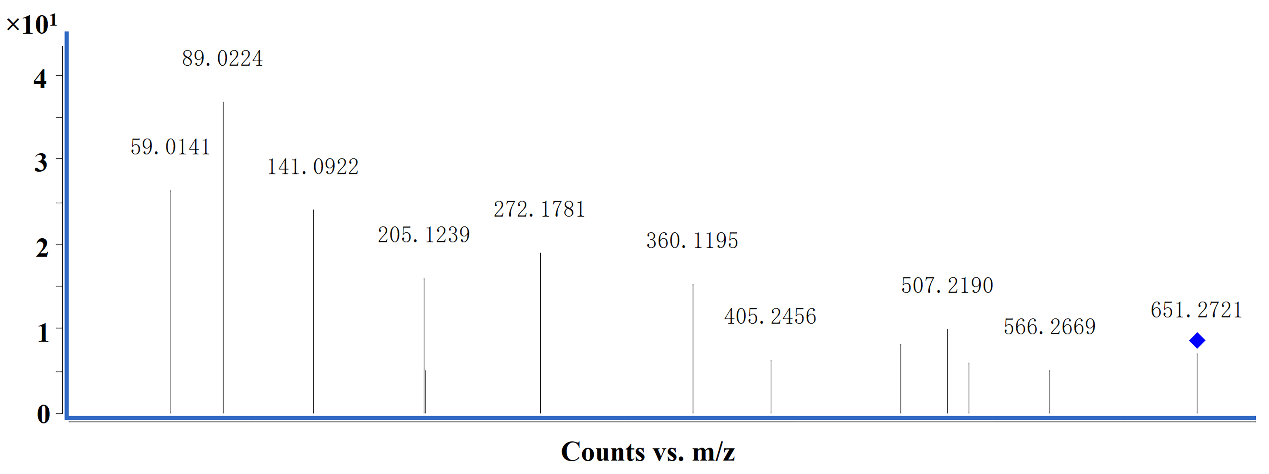


27


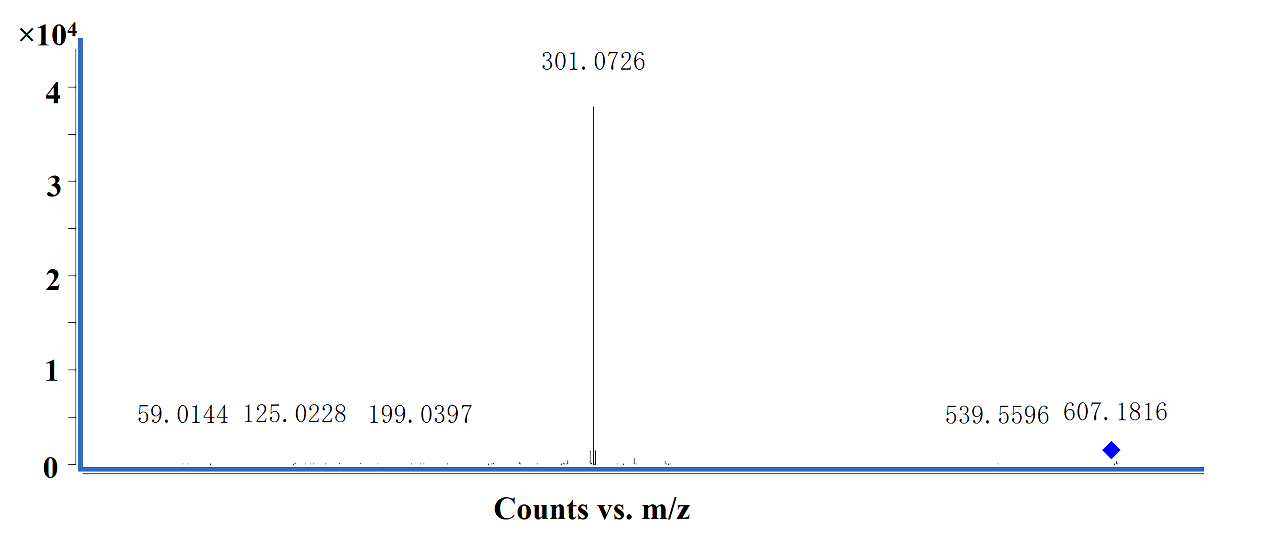


28


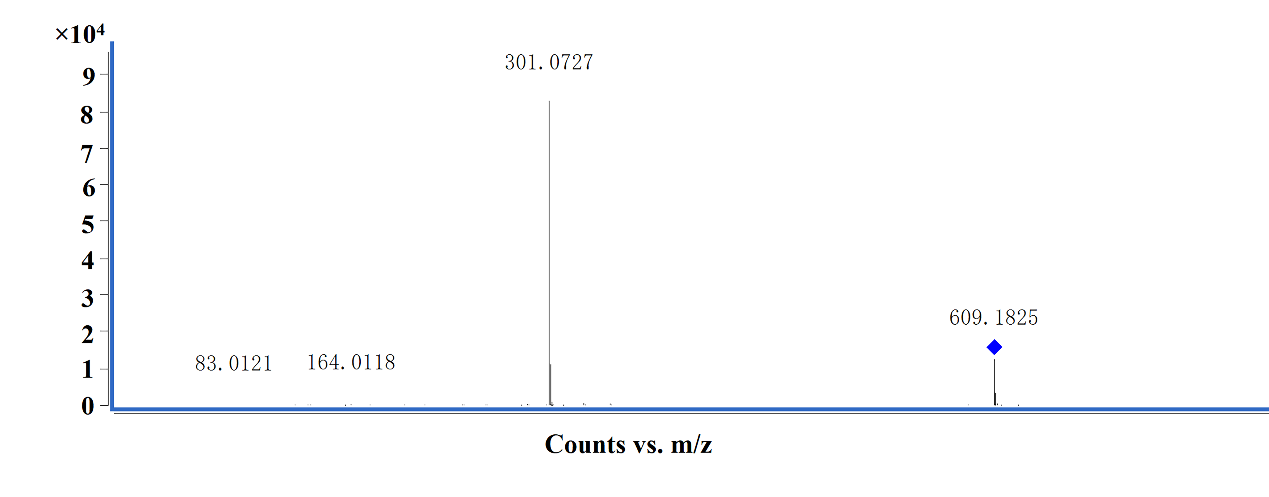


29


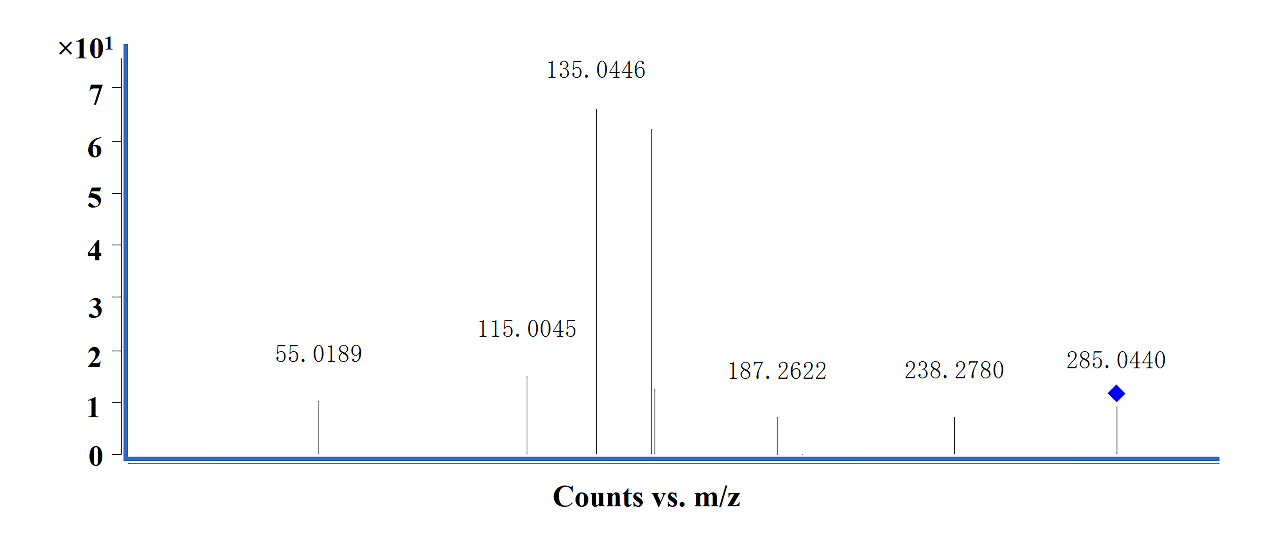


30


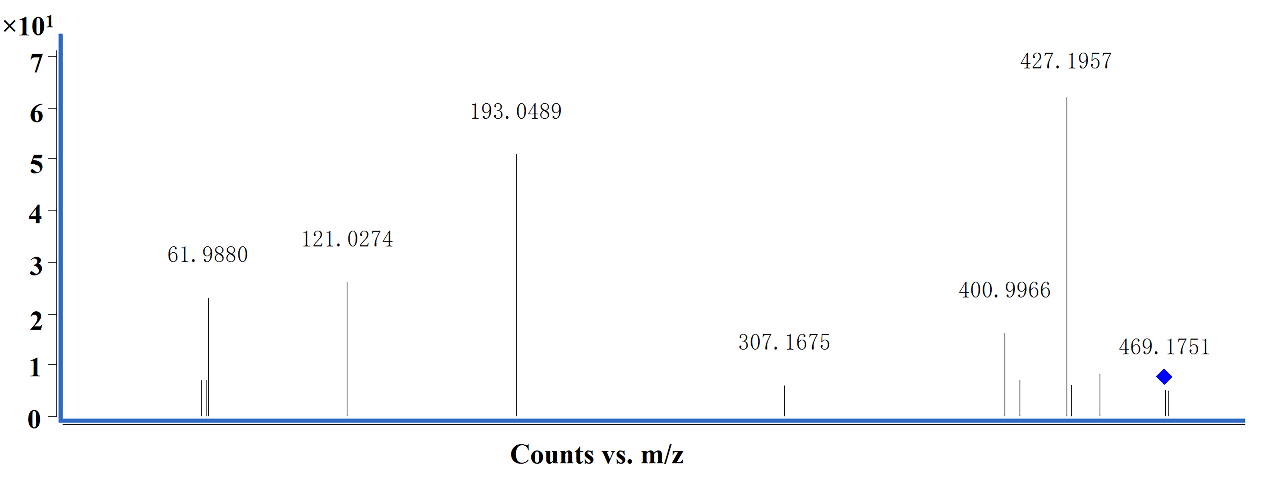


31


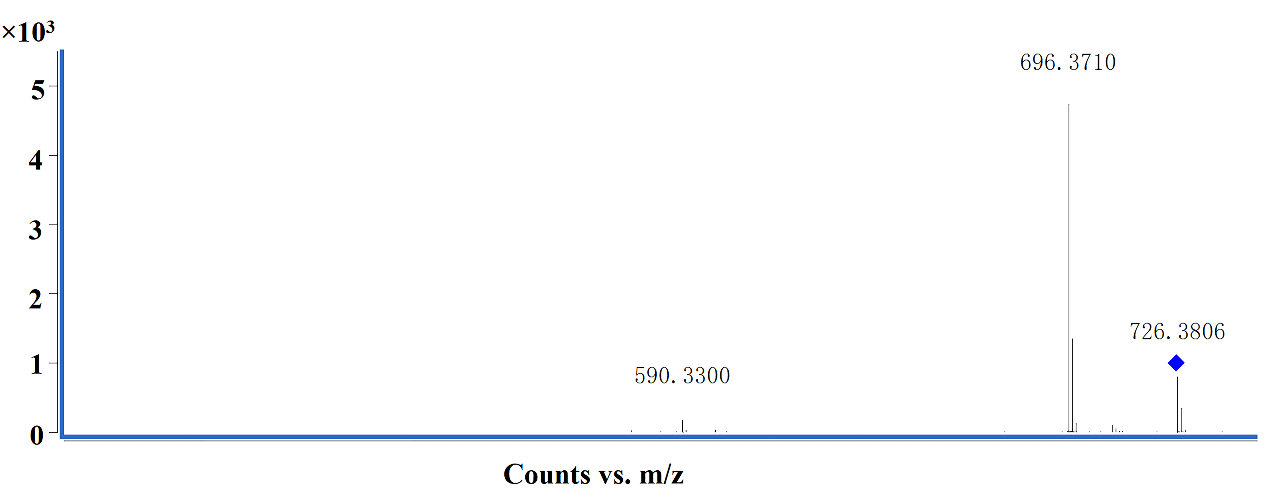


32


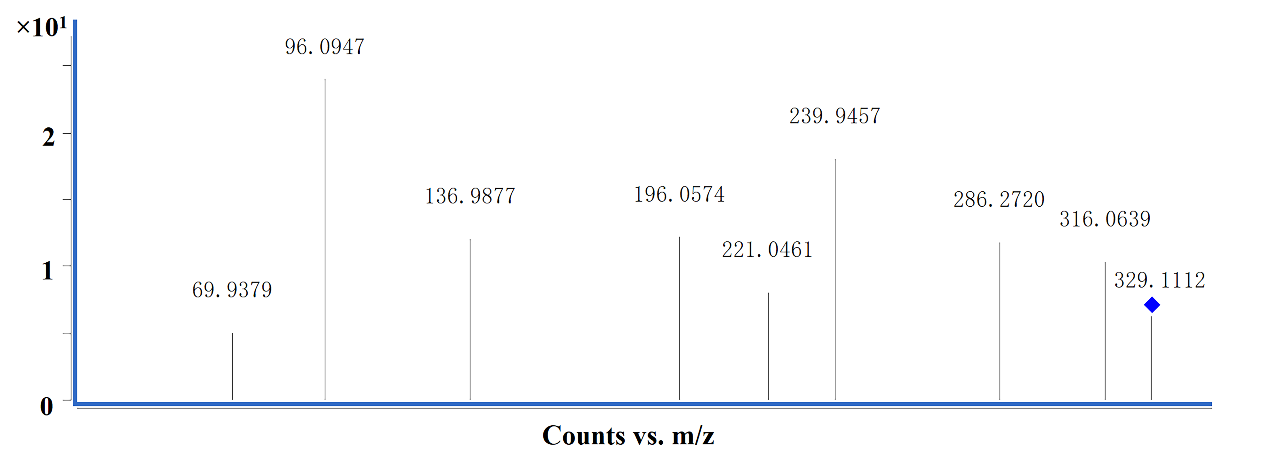


33


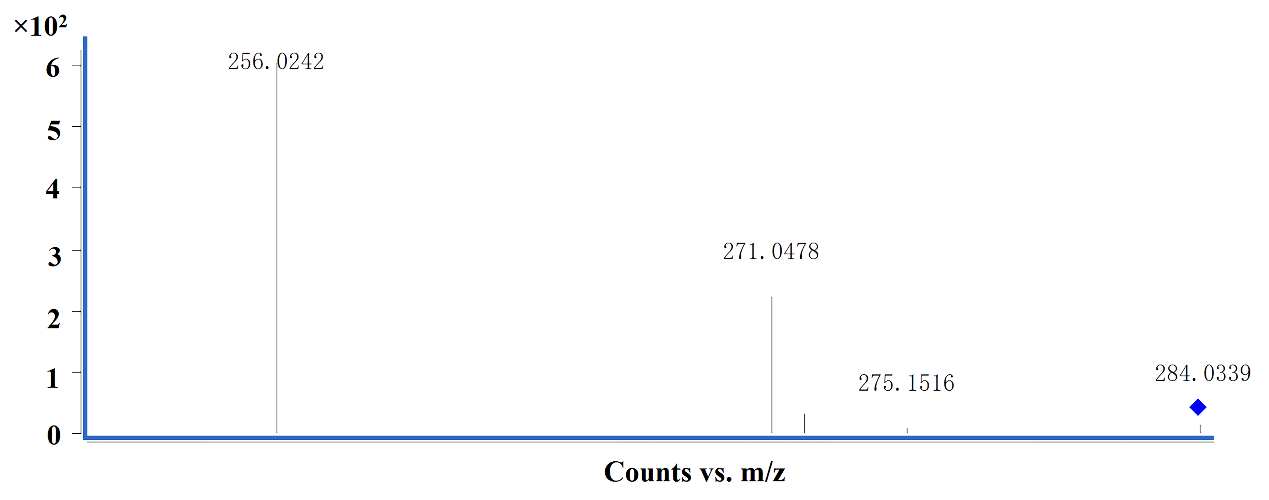


34


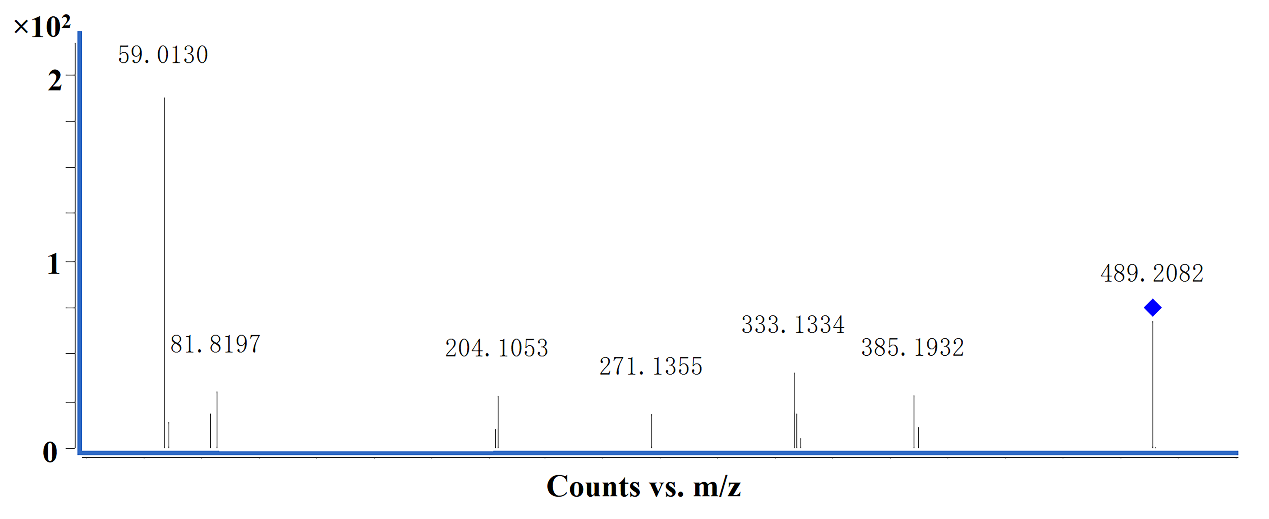


35


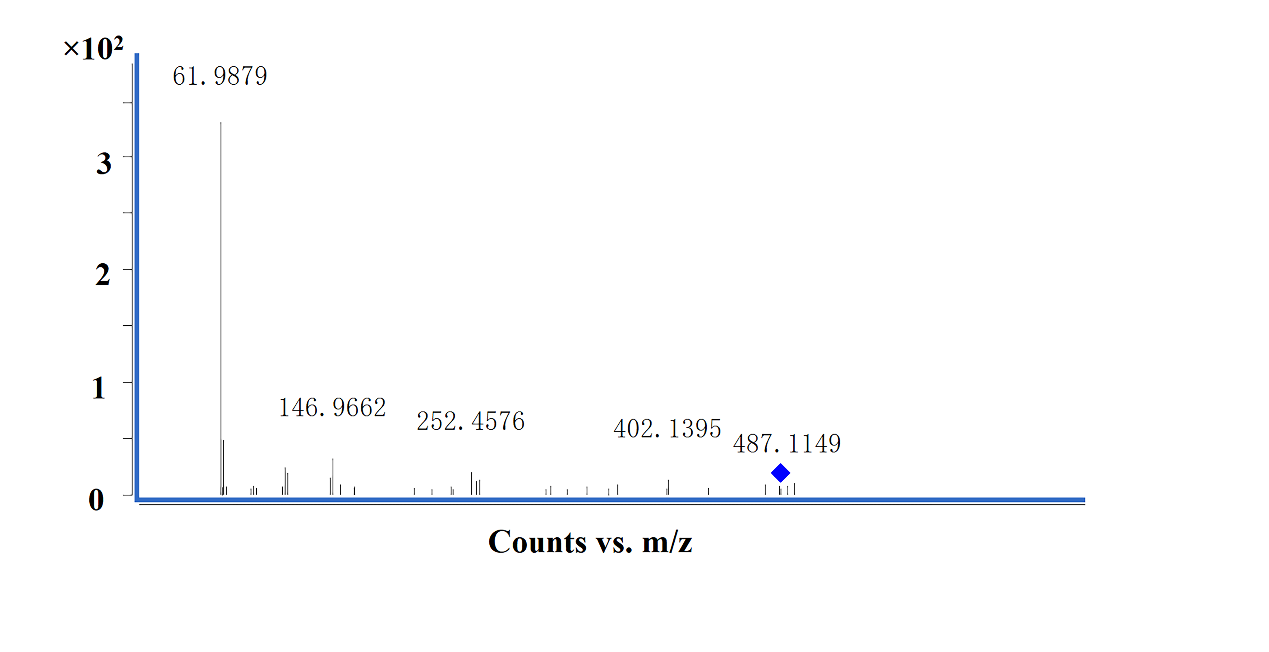


36


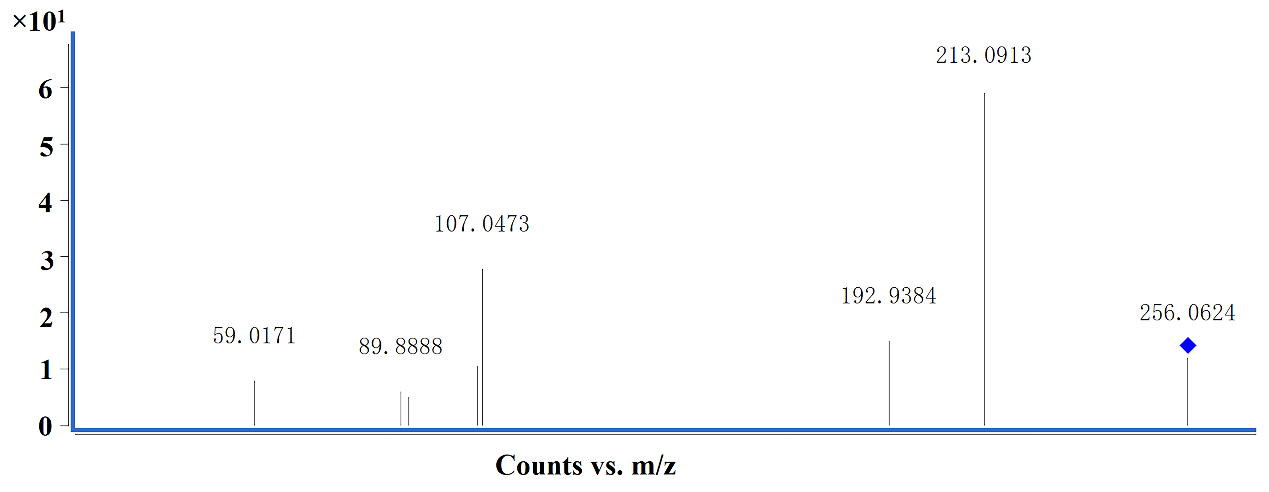


37


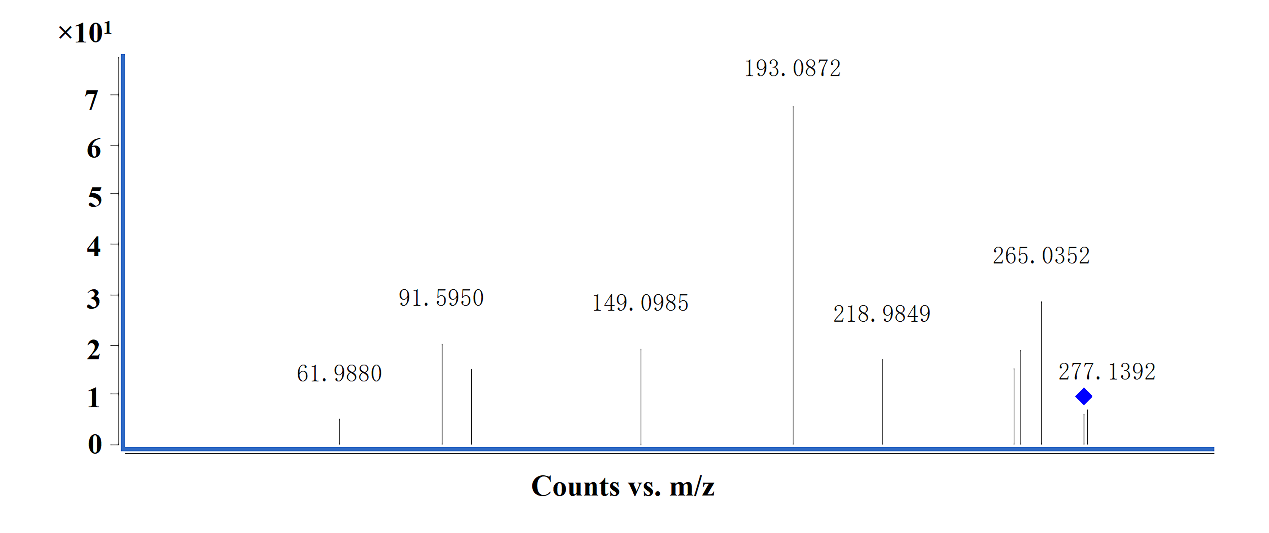


38


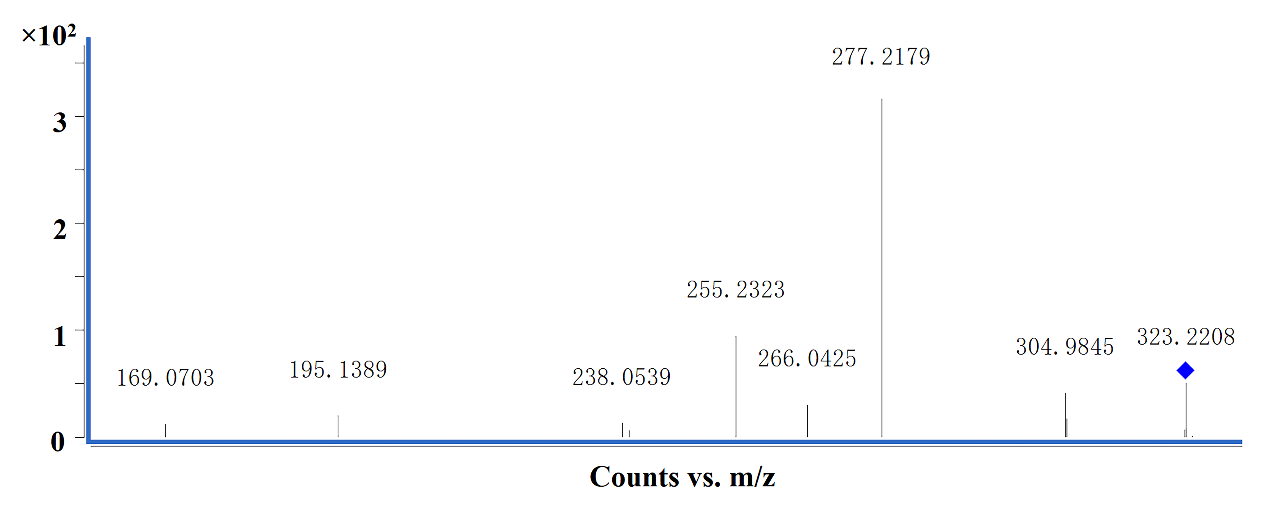


39


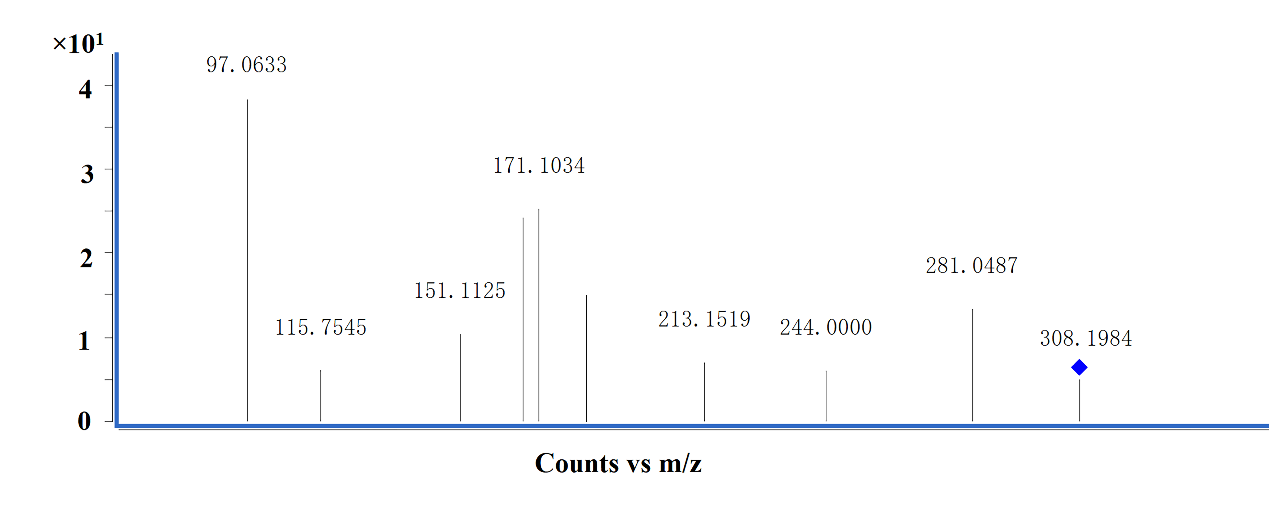


40


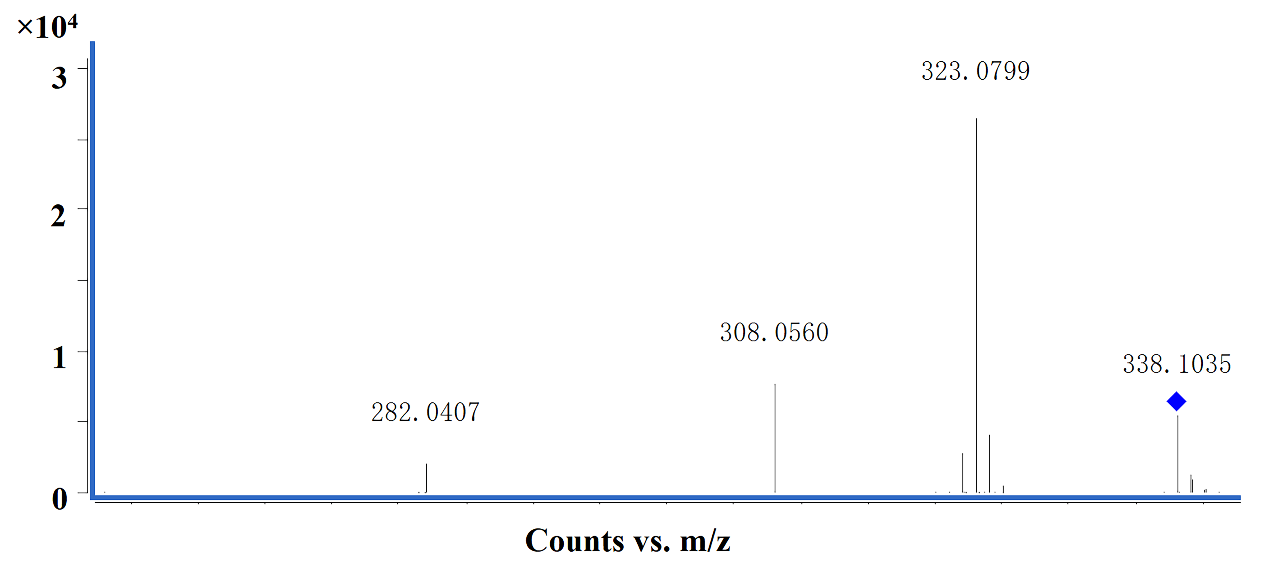


41


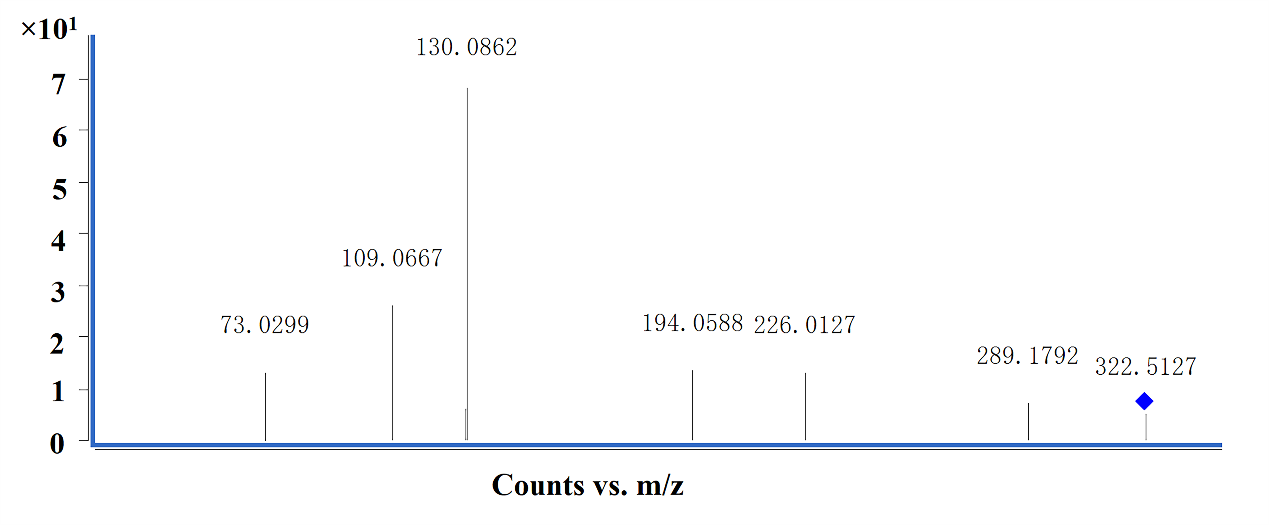


42


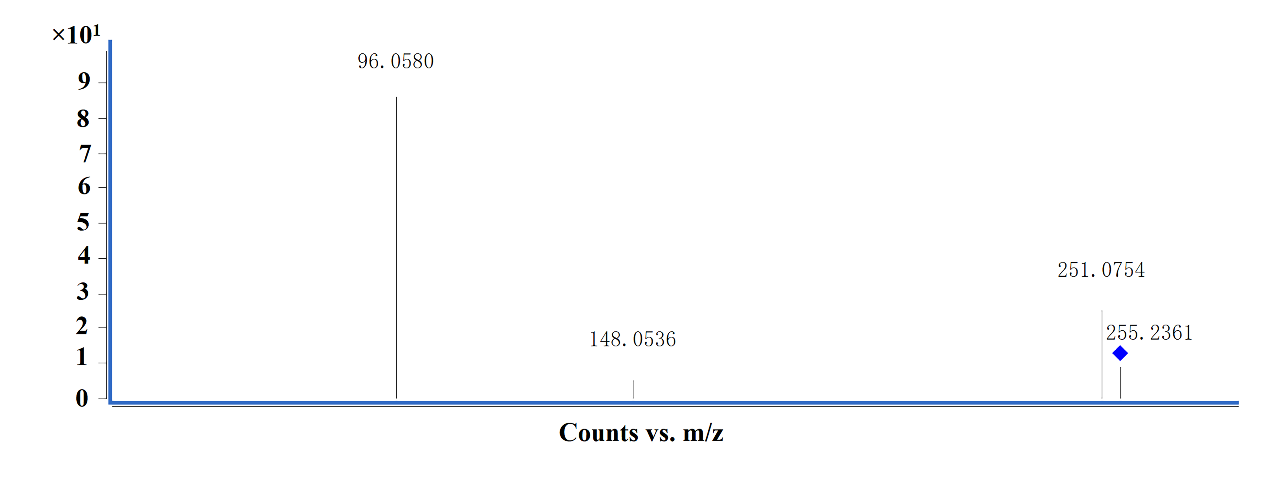


43


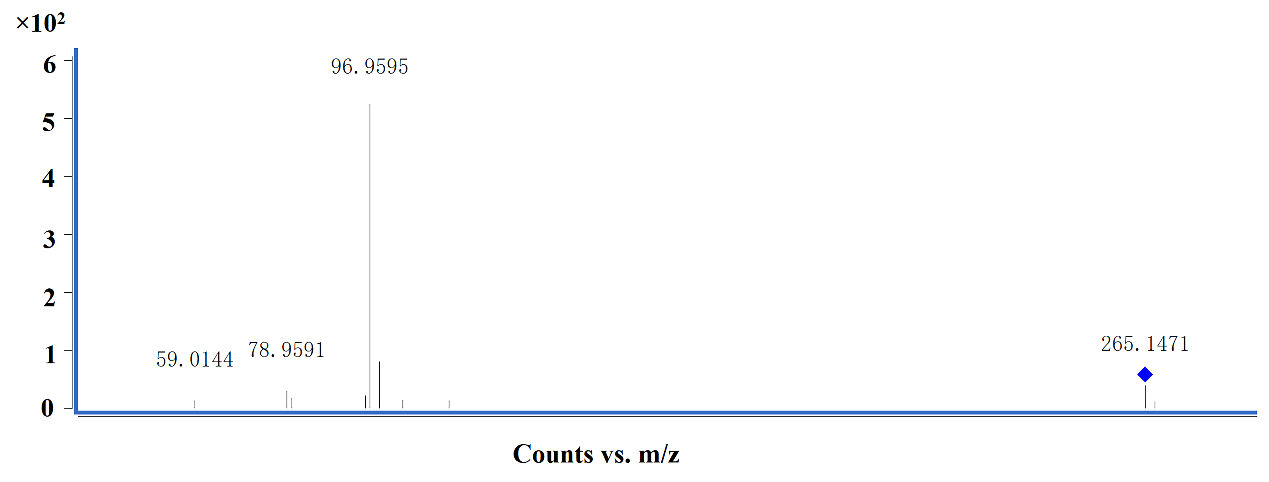


44


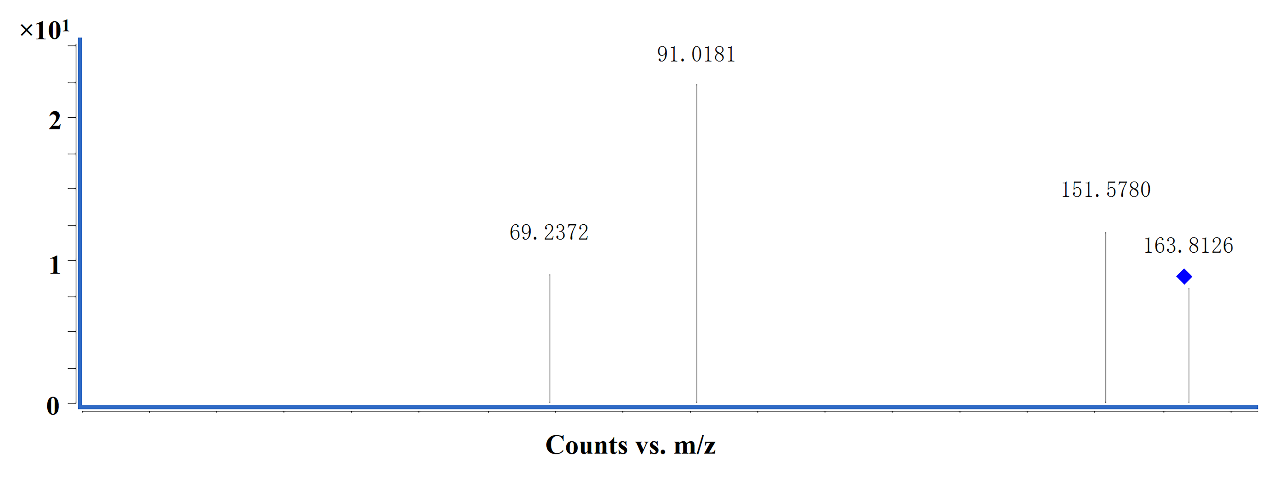


45


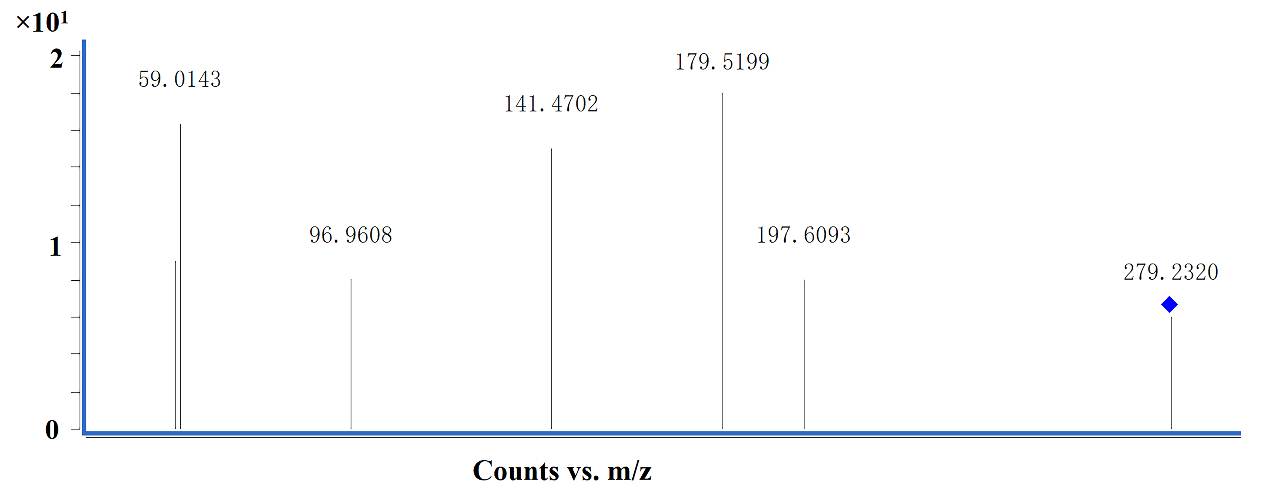


46


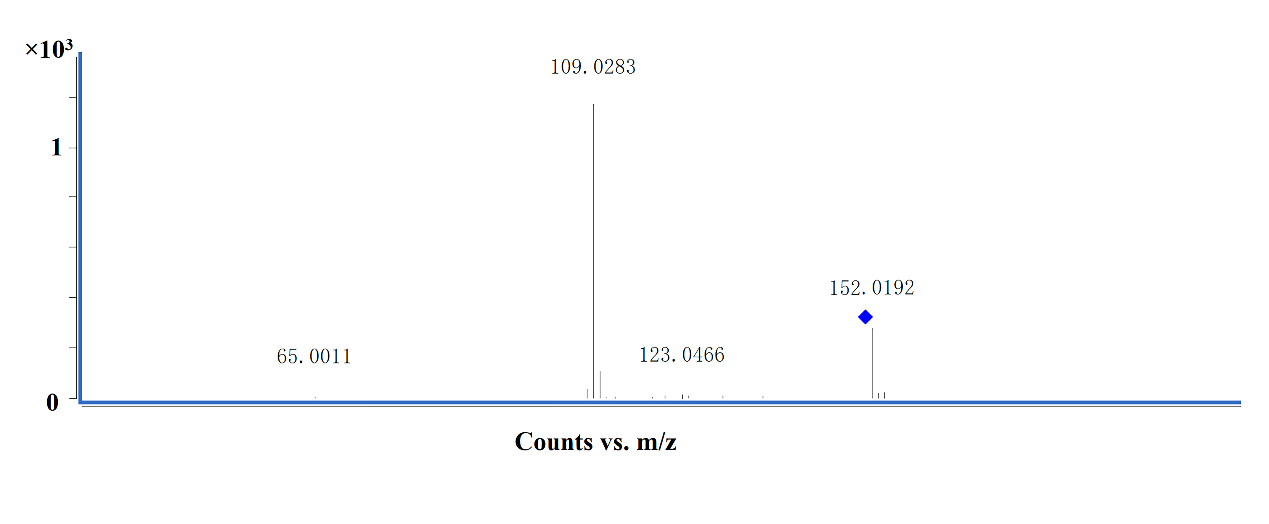


47


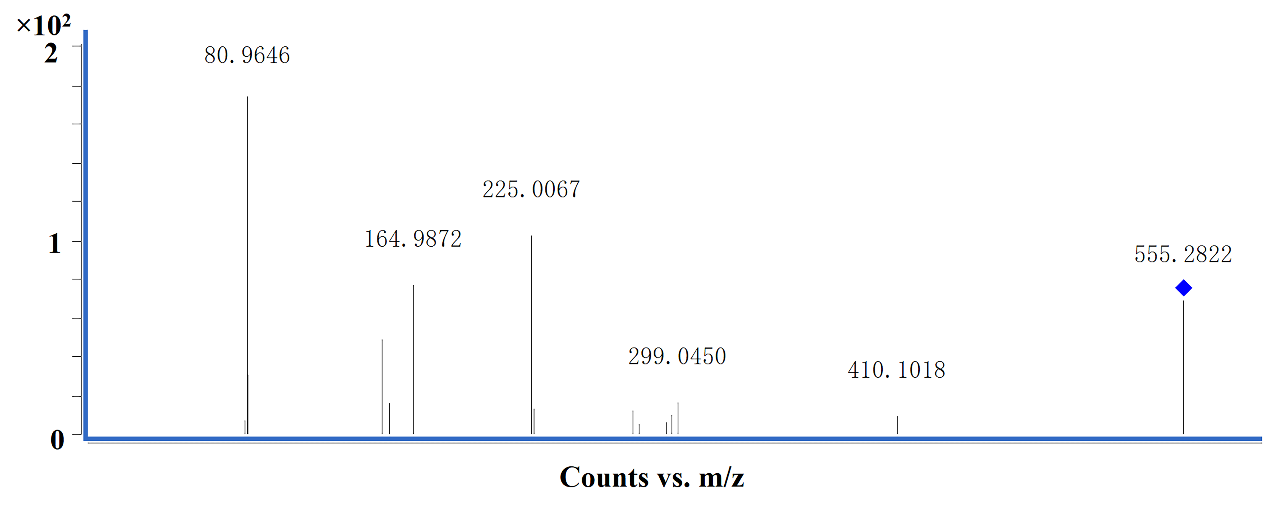

Supplement: Supplementary file 1 [file Data_Sheet_1.DOCX]
